# Seeing the Unseen: RCPNet’s Dual Strategy for Occluded and Similar-Color Sweet Persimmon Detection in Dense Canopies

**DOI:** 10.3390/plants15162490

**Published:** 2026-08-17

**Authors:** Shilin Li, Lili Sun, Chaoyi Wu, Wenyang Zang, Shujuan Zhang, Fuzhong Li

**Affiliations:** 1 Faculty of Software Technologies, Shanxi Agricultural University, Jinzhong 030800, China; 2 College of Agricultural Engineering, Shanxi Agricultural University, Jinzhong 030800, China

**Keywords:** object detection, automated picking, contextual information, occlusion detection, near-color recognition

## Abstract

In complex orchard environments, sweet persimmons tend to grow in dense clusters and display similar coloration across different maturity stages, leading to heavy occlusion and poor inter-class color discriminability. To address these challenges, this paper presents RCPNet, a detection network tailored for such field conditions. The model integrates a Rectangular Self-Calibration Module (RCM) and a Context Feature Calibration Gating (CFCG) module. RCM strengthens axial context capture, while CFCG improves feature calibration; together they reduce local feature ambiguity and help reconstruct missing information in occluded regions. For distinguishing fruits at different ripening stages that share similar colors, a Parallelized Patch-aware Attention (PPA) detection head is adopted. By leveraging self-attention and multi-branch strategies, this head suppresses feature degradation and notably enhances sensitivity to color contrast. Experiments on sweet persimmon images show that RCPNet improves mean Average Precision (mAP) by 2.7 percentage points and mAP@0.5:0.95 by 3.7 percentage points over the baseline, reaching 93.6% detection accuracy for immature fruits. Ablation studies and comparisons with mainstream detectors indicate that the proposed model, though slightly heavier than lightweight detectors of analogous capacity, surpasses the accuracy of a larger small-scale counterpart and exhibits satisfactory robustness. Strong performance on a self-collected flat jujube dataset further confirms its generalization ability. The method delivers highly accurate detection for occluded and near-color fruits, providing technical support for precise fruit recognition and automated picking.

## 1. Introduction

Persimmon (Diospyros kaki Thunb.) is a traditional medicinal and edible plant in China, with its cultivars primarily classified at harvest into two groups—sweet persimmons and astringent persimmons—based on natural deastringency levels. With the continuous expansion of orchard planting areas and the development of smart agriculture, automated picking has become imperative. However, the detection of field sweet persimmons faces two major challenges: boundary ambiguity and missed/false detections under dense occlusion, and similar-color confusion between different ripeness stages. Specifically, (1) the color change is not obvious during ripening, making it difficult to judge the maturity; and (2) the clustered growth of the fruits results in boundary ambiguity and significant occlusion, elevating missed detection rates. Therefore, there is an urgent need for an effective object detection algorithm to detect field sweet persimmon.

Dense prediction problem. In complex agricultural environments, the clustered growth of fruits and the resulting issues of occlusion and overlap further exacerbate the loss of information in occluded areas, due to certain areas being invisible or having blurred boundaries. Despite recent progress, most existing methods for occlusion handling are developed and tested on datasets where occlusion patterns are relatively structured. In dense fruit canopies, standard approaches face two key limitations. First, they suffer from insufficient contextual extraction. Methods that rely on local convolutions or unstructured self-attention cannot capture the multi-scale, structured context needed to separate individual fruits from a complex canopy. Second, they lack a contextual calibration mechanism. Even when broader context is gathered, there is no way to check whether the attended context is semantically consistent with the query fruit. This weakness is especially critical in similar-color detection tasks. To address this issue, there are generally two approaches [1,2,3]. The first method involves utilizing specialized convolutions to enhance the model’s capability in handling irregular occluded regions [4]. For example, Zhu et al. [5] incorporated Deformable Convolution (DCN), achieving an average grape-picking success rate of 87.4%. Similarly, Guo et al. [6] redesigned the backbone network through DCNv4 to enhance the model’s capability in detecting irregularly shaped objects and small-scale targets. Meanwhile, Xiong et al. [7] utilized a dynamic convolution approach to enhance feature representation by reconstructing missing features within occluded regions. The second method leverages contextual information to augment occlusion awareness, inferring the occluded parts based on the visible sections [8,9]. To integrate contextual information at different scales, Li et al. (2025) [10] proposed a multi-scale perception module for grading the severity of kiwifruit leaf disease, achieving an accuracy of 89.57%. However, uncalibrated contextual features were prone to omissions. Yang et al. [11] proposed a dynamic context-aware aggregation strategy, improving the detection accuracy by one to seven percentage points. In addition, the attention mechanism can be integrated to calibrate and aggregate contextual feature information. Guo et al. [12] proposed a lightweight weed detection model incorporating joint global context. This model enhances fused feature learning by introducing global contextual information via a style refinement module. Therefore, leveraging contextual information to enhance the feature representation of occluded regions is a feasible direction worthy of in-depth exploration. Beyond agriculture, occlusion is a fundamental challenge in remote sensing as well. NIRNet [13] handles cloud corruption by simulating it during training, but agricultural occluders such as leaves are texturally complex and a similar color to fruit, motivating our affinity-based recalibration over synthetic augmentation. Unc-SOD [14] models predictive uncertainty to suppress false positives. This parallel suggests that context-guided attention recalibration generalizes beyond agriculture.

Low color discriminability. In the field of target detection for agricultural products, it encompasses not only the issue of similar colors between fruits and the background, but also the issue of similar colors among fruits at different maturity stages [15]. To avoid recognition confusion caused by similar-color features, Sun et al. [16] introduced a focal multi-head self-attention mechanism for green apples to achieve aggregation of local and global information. Since green fruit is similar in color to the background, Fu et al. [17] constructed feature information from different angles and transmitted local key information to deeper layers, achieving an accuracy rate of 82.2%. Using the Convolutional Block Attention Module (CBAM), Zhang et al. [18] captured relevant channel and spatial information, enhancing green citrus image feature extraction. Zhao et al. [19] incorporated deformable convolution and the LSC attention module to fully extract the feature information of green fruits. Huang et al. [20] achieved a green pepper detection accuracy of 82.2% in complex scenarios using a reversible dual-pyramid architecture designed for enhanced multi-level feature extraction. In addition to the green bell peppers, green apples, and green citrus fruits mentioned above, green fruits include those with unique forms, such as cucumbers and bitter gourds. For similarly colored fruits with distinctive shapes, Zhao et al. [21] added a key point recognition branch and a mask generation branch to identify cucumbers against a similar-colored background, achieving an accuracy rate of 94%. Jiang et al. [22] employed dynamic snake convolution to extract morphological features from the curved and elongated structures of bitter gourds. In addition, there is the recognition of small targets [23,24] with similar colors, such as tea buds and weeds. Liu et al. [25] designed a three-branch attention mechanism to enhance the features of tea bud objects and utilized self-calibrating group convolution to better extract contextual features, achieving mAP values of 87.04% and 94.5%, respectively. Sweet persimmon maturity recognition is essentially a fine-grained classification task with weak inter-class differences, and therefore places higher demands on feature discriminability. Unlike the widely studied fruit–background color-similarity problem, inter-maturity near-color detection presents a distinct and harder challenge: the objects belong to the same semantic class, their chromatic distributions heavily overlap, and the discriminative cues often reside in extremely subtle textural or size variations. This problem has received comparatively little dedicated attention in the agricultural computer vision literature.

Motivated by these specific limitations, our RCPNet framework introduces three complementary modules. (1) The RCM module captures axial context information to enhance handling of dense scenes, infers occluded objects, and compensates for missed detections. (2) The CFCG module, incorporating a spatial attention gating mechanism, conducts context feature calibration to mitigate weight mismatch in contextual information, enhancing feature perception in occluded regions. (3) The PPA module is utilized to enhance local structure perception and suppress color interference.

## 2. Materials and Methods

### 2.1. Construction of Dataset

**Figure 1:**
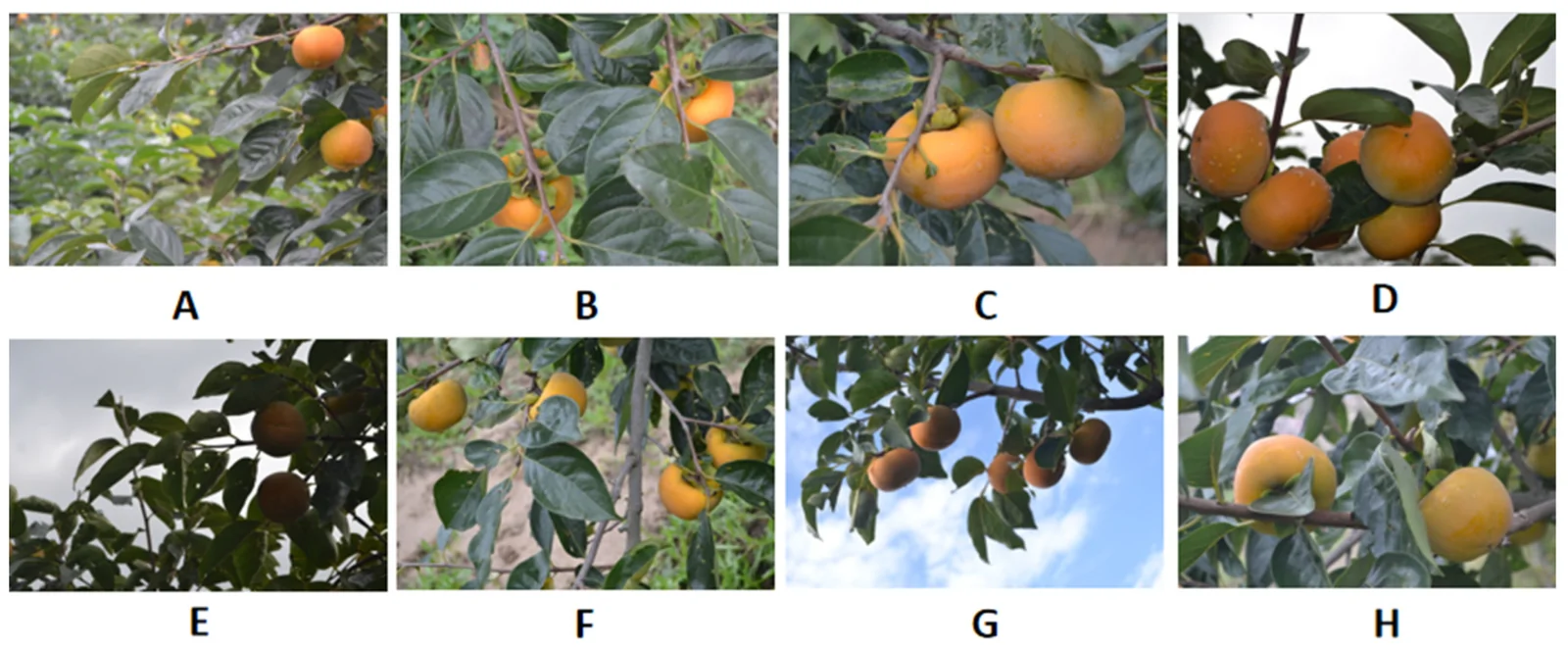
Partial sample data. (**A**) Front lighting; (**B**) leaf obstruction; (**C**) branch obstruction; (**D**) clustered growth; (**E**) back lighting; (**F**) ground background; (**G**) sky background; (**H**) canopy background.

In this study, ‘Yang Feng’ sweet persimmon from Wanrong County, Yuncheng City, Shanxi Province, China, was chosen as the experimental subject. The sweet persimmon images were taken from 7 to 17 October 2024, and the acquisition equipment for the images was a Canon EOS 650D camera (Canon Incorporated, Tokyo, Japan). Images were captured between 7:00 and 12:00 and between 14:00 and 19:00 at a shooting distance of 50–80 cm. Sweet persimmons were photographed from multiple angles under front-lighting and back-lighting conditions, incorporating diverse backgrounds (sky, ground, canopy) and obstructions (branches, leaves, fruits). The sample data are shown in Figure 1.

A total of 7570 images with a resolution of 3456 × 2304 pixels were acquired and stored in JPEG format. After dehazing the original images and enhancing the images in the training subset, the final dataset consisted of 8701 images. To optimize computational efficiency, all images were resized to 512 × 341 pixels using bicubic interpolation. All images were manually annotated using LabelImg 1.8.6 (Tzutalin, Canada), yielding XML files that store the object category labels and bounding box coordinates. Fruits in a visually ambiguous ripening-transition stage that could not be confidently assigned by the annotator were left unlabeled. These unlabeled fruits were excluded from both training supervision and evaluation metrics. No fruit was forcibly assigned to a category against the annotator’s judgment. The dataset was partitioned into mutually exclusive training, validation, and test subsets at ratios of 80%, 10%, and 10% through randomized sampling. A detailed breakdown of the sample distribution is provided in Table 1.

In addition, we employed a flat jujube dataset that also presented color similarity and occlusion challenges, along with small object challenges, to improve the generalizability of our findings and verify the model’s generalization. The dataset was collected from September 15 to 28, 2023, in Linyi County, Shanxi Province, using a Nikon D3100 (Nikon Corporation, Tokyo, Japan) camera and an Honor 8X Max smartphone. It contained 9525 images in total.

### 2.2. Improvement in the RCPNet

#### 2.2.1. Rectangular Self-Calibration Module

**Figure 2:**
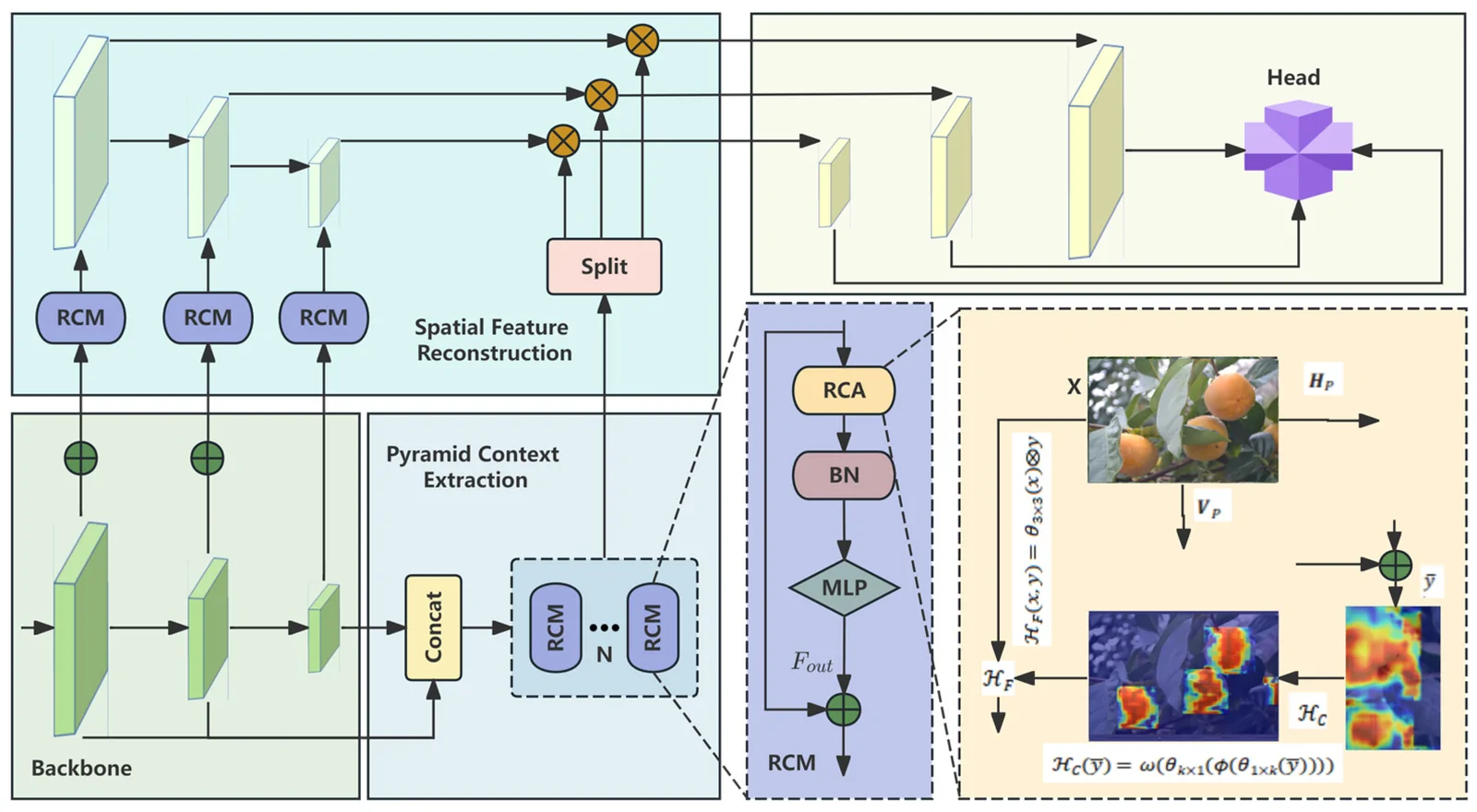
The structural diagram of the RCM. The module consists of RCA, BN, and MLP. RCA explicitly models the rectangular region and calibrates the attention shape.

Current lightweight models, hampered by inadequate feature representations, frequently struggle with precise contour delineation and discriminative category identification for target objects. Consequently, these deficiencies manifest as imprecise boundary segmentation and erroneous category predictions. The Rectangular Self-Calibration Module (RCM) is purpose-built for spatial feature reconstruction and pyramid context extraction. The module employs horizontal and vertical pooling operations to extract the axial global context, yielding two orthogonal axis vectors. These vectors are combined via element-wise summation to formulate a rectangular attention region. To refine the spatial alignment of this region with foreground features, a shape self-calibration mechanism is incorporated, which utilizes large-kernel strip convolutions. Furthermore, an attention feature fusion operation enhances local detail representation. Collectively, the RCM enhances foreground focus for spatial feature reconstruction while simultaneously facilitating effective axial global context capture for pyramid context extraction. The architecture of the RCM is shown in Figure 2.

The RCA mechanism employs horizontal and vertical pooling operations to extract the axial global context along both spatial dimensions, yielding two orthogonal axis vectors. Through broadcast addition of these vectors, RCA effectively models a rectangular region of interest (ROI). Subsequently, a shape self-calibration mechanism refines the spatial alignment of this ROI with foreground objects. This calibration leverages two large-kernel strip convolutions, applied in a decoupled manner in the horizontal and vertical directions. The weights of the strip convolutions are learnable, and the shape of the highlighted area is changed by two strip convolutions with different weights. By training, this function can learn the appropriate weights to adjust the rectangular area to the foreground object. Specifically, horizontal strip convolution adjusts the ROI’s shape row-wise, enhancing its horizontal correspondence with the foreground. Following standard processing steps (Batch Normalization and ReLU activation), vertical strip convolution similarly calibrates the column-wise structure. This decoupled application of directional convolutions enables the mechanism to adapt flexibly to arbitrary object geometry. Meanwhile, the RCM incorporates a Multilayer Perceptron (MLP) to further enrich feature representation capabilities. Furthermore, the RCA effectively models rectangular regions of interest and refines feature focus towards foreground elements via its shape self-calibration mechanism. This self-calibration mechanism is mathematically formulated as follows:

(1) $$H_{C}(\bar{y})=\omega (\theta_{k\times 1}(\phi (\theta_{1\times k}(\bar{y}))))$$

where θ indicates the large-kernel strip convolution, k indicates the kernel size of the strip convolution, ϕ indicates the BN followed by the ReLU function, and ω indicates the sigmoid function.

Additionally, a feature fusion module integrates the attention features with the original input features. Specifically, a 3 × 3 depth-wise convolution is applied to the input features to enhance local detail extraction. The calibrated attention features are then weighted onto these refined input features via the Hadamard product, with the following mathematical expression:

(2) $$H_{F}(x,y)=\theta_{3\times 3}(x)⨂y$$

where θ_3×3_ indicates the depth-wise convolution with a 3 × 3 kernel, y is the attention feature obtained in the previous step, and ⨂ is the Hadamard product.

Specifically, BN and MLP are incorporated following RCA to enhance feature discrimination. Subsequently, a residual pathway is employed to further promote feature reuse. This process is formally expressed as:

(3) $$Q_{out}=\varepsilon (H_{F}(x,H_{C}(H_{p}(x)⊕V_{p}(x))))+x$$

where ⊕ indicates broadcast addition, HP and VP represent horizontal pooling and vertical pooling, and ε refers to BN and MLP.

Standard contextual attention often relies on square receptive fields, which are suboptimal for the elongated, irregular shapes formed by clustered persimmons. RCM explicitly employs rectangular (axial) self-calibration, enabling the model to capture long-range dependencies along both the horizontal and vertical axes of clusters. This design is directly motivated by the observation that occlusion patterns in our orchards are heavily directional.

#### 2.2.2. Context Feature Calibration Gating Module

Context mismatch primarily arises from the uniform processing inherent in conventional context modeling approaches, which lack adaptability to diverse inputs. Specifically, prevalent context aggregation methods generate non-adaptive contextual features per pixel, neglecting intrinsic variations in context requirements. To address this limitation, the CFCG module employs a pooling-based strategy. It dynamically adapts context per pixel by matching individual pixel representations with pooled context features. Unlike conventional context aggregation schemes that apply static mechanisms per pixel, the CFCG module generates pixel-wise contextual representations. These representations are input-dependent and vary across spatial locations.

The CFCG module integrates a cascaded pyramid pooling block within a self-attention framework, introducing a context refinement strategy to customize multiscale contextual representations per pixel while reducing computational complexity. Diverging from standard self-attention’s pixel-to-pixel similarity, CFCG calculates pixel-to-context similarity to aggregate relevant semantic contexts per spatial location, enabling context feature calibration. However, since pooling operations inherently exhibit bias toward large-scale patterns, their uniform distribution across all locations dominates the representation of smaller features, inducing over-smoothing. To address this, the Context Re-calibration Block (CRB) incorporates adaptive mechanisms to selectively acquire local contextual information, enhancing the structural delineation of large objects while preserving fine-grained spatial details.

Consider the occluded scenario. For a corrupted query q, the raw dot-product similarity x_q_^T^W_k_x_k_bg_ might be high for a background leaf key k_bg. However, the context prior C_q_—derived from the neighborhood pool—correctly encodes “fruit” texture, not “leaf” texture. Consequently, the affinity Φ between the fruit prior and the leaf feature will be low. This gates down the mismatched attention:

(4) $$\Phi_{{q,k}_{bg}}=\sigma (c_{q}^{T}W_{c}x_{k_{bg}})$$

(5) $$\Phi_{{q,k}_{fruit}}\gg \Phi_{{q,k}_{bg}}$$

where W_c_ is a learnable projection that maps features into the affinity space, and σ is a non-linear activation. Intuitively, Φ_q,k_ quantifies the probability that key k belongs to the same semantic entity as the query region, based on the robust context prior. Simultaneously, for a genuine fruit key k_fruit, even if partially occluded, its feature x_k_fruit_ retains some fruit characteristics. The attention on true same-object pixels is therefore preserved and amplified.

**Figure 3:**
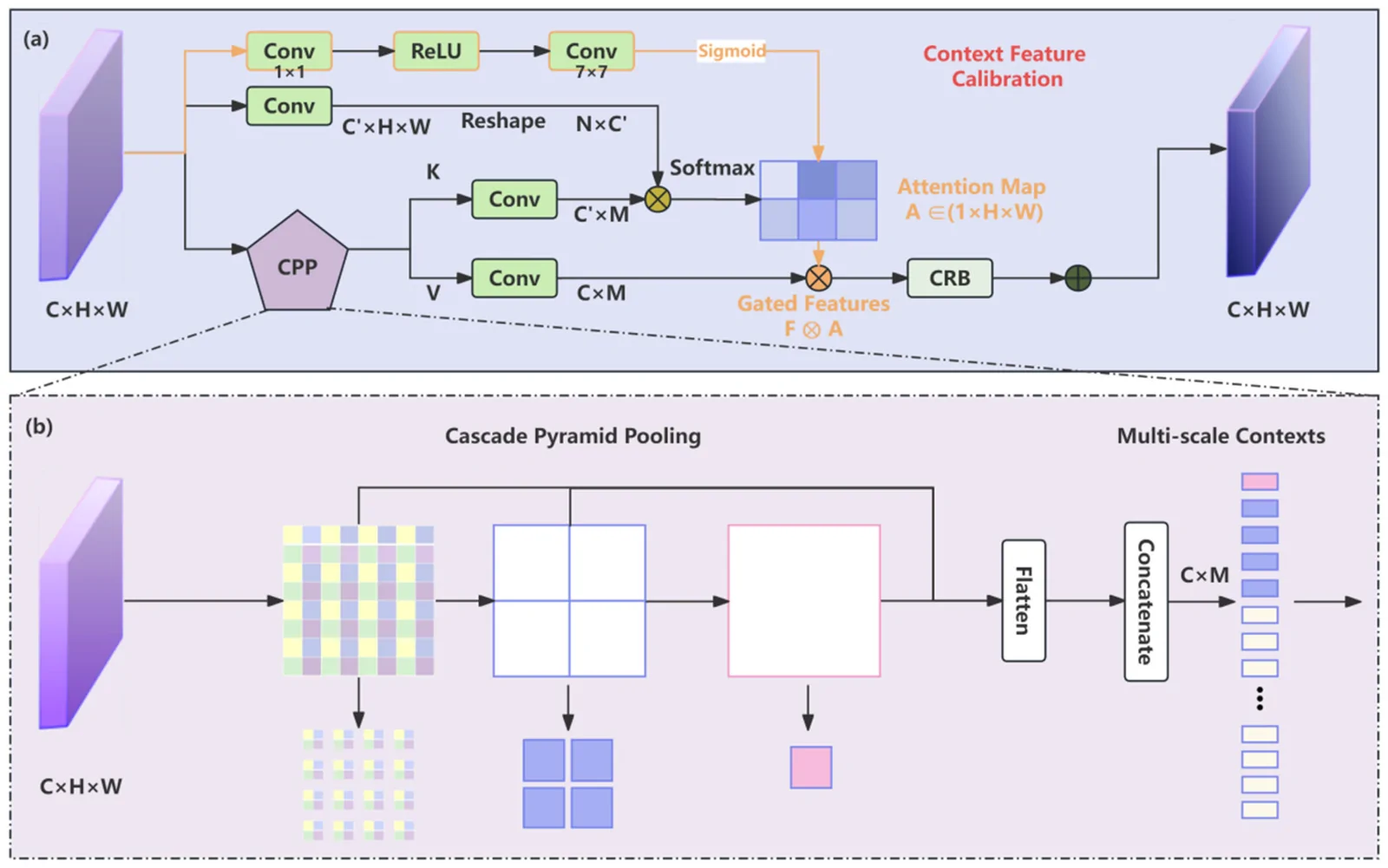
Schematic diagram of the CFCG module. (**a**) The implementation procedure of the CFCG module. (**b**) Demonstration of our cascade pyramid pooling layer.

In summary, the pixel-context affinity matrix acts as a semantic consistency filter that calibrates raw self-attention using a locally pooled, occlusion-robust context. The pooling operator supplies resilience against pixel-level corruption, while the affinity matrix mathematically enforces that attention can only be directed to keys that are semantically aligned with the robust context. This jointly resolves the weight mismatch problem. The specific structure is shown in Figure 3.

As depicted in Figure 3a, given an input feature map X ∈ R^C×H×W^, we first apply a 1 × 1 convolutional layer to generate a reduced-dimensional feature F^in^ ∈ R^C′×H×W^, where C′ ≪ C (by default, C′ = 32, C = 256). We incorporated a spatial attention gating mechanism (orange section) to suppress background clutter that was not present in the original CSFCN block, which is critical for distinguishing fruits from the dense canopy. In parallel, a spatial attention gate applies a 1 × 1 convolution with channel reduction ratio r = 16, followed by ReLU, a 7 × 7 convolution with a single output channel, and sigmoid activation to generate a spatial attention map A ∈ R^1×H×W^. The final output is F_out_ = F_fused_ ⊗ A + F_in_, where ⊗ denotes element-wise multiplication. This gating mechanism adaptively suppresses background clutter while preserving fine-grained fruit features.

As in Figure 3b, the Cascaded Pyramid Pooling (CPP) block efficiently propagates hierarchical features by reusing pooling results from preceding layers, thereby minimizing computational redundancy. Then, the CPP block aggregates these features to yield multi-scale contextual representations P ∈ R^C×M^. Specifically, we define the output height n of the pooling layer within the set [1, 2, 3] (typically, the output width equals the height; by default, n = [1, 2, 3, 6]). Standard pooling operations apply a homogeneous spatial grid, potentially introducing distortion in the pooled features when the input aspect ratio deviates from 1. To mitigate this, we maintain the input aspect ratio in the pooling output size.

Afterward, F is reshaped and transposed to R^N×C′^. The matrix product between the transformed F and K is then computed, followed by a softmax layer, yielding the pixel-context affinity matrix ω ∈ R^N×M^:

(6) $$\omega =\frac{exp(F_{i}\cdot K_{j})}{\sum_{j=1}^{M}exp(F_{i}\cdot K_{j})}$$

where ω_i,j_ represents the affinity between ith pixel F_i_ and jth context K_j_.

Finally, the matrix product between V and ω^T^ is computed, yielding the calibrated semantic context N ∈ R^C×N^. This tensor is then reshaped to N ∈ R^C×H×W^. The resulting calibrated context N is propagated through the CRB to generate the refined context N′.

CFCG incorporates a gating mechanism that adaptively decides whether and how much to calibrate each position. This is fundamentally different from fixed-map recalibration in SE or CBAM. The gate suppresses ambiguous features from occluded areas while preserving clean foreground signals.

#### 2.2.3. Parallelized Patch-Aware Attention Module

**Figure 4:**
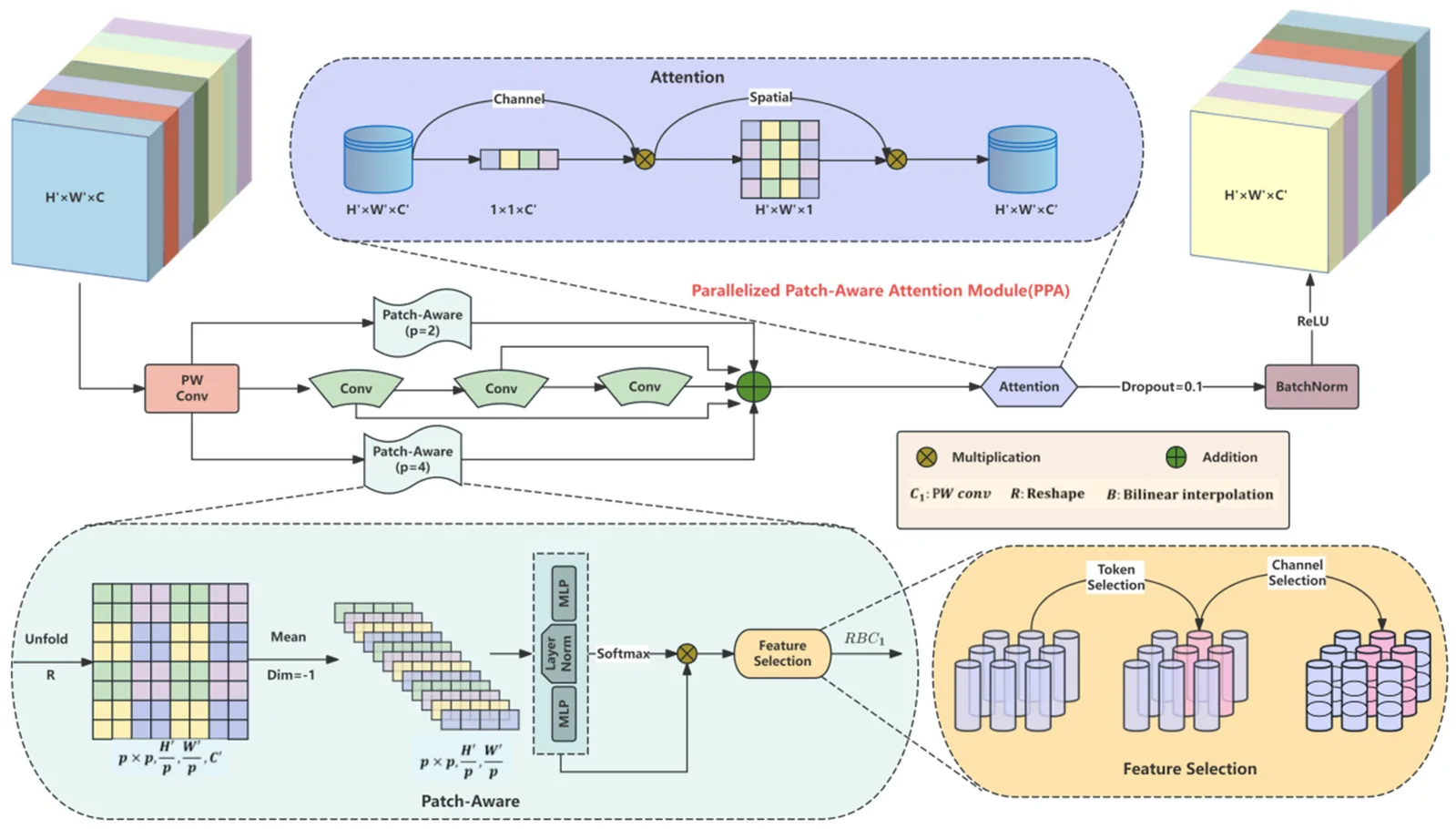
The structural diagram of the PPA module. The ‘p’ parameter in Patch-Aware is 2 and 4, representing the local branch and the global branch, respectively.

To address the aforementioned two detection challenges (feature loss and low discriminability in complex backgrounds), this paper introduces the PPA module, with an attention mechanism in the detection head. The core advantage of PPA lies in its multi-branch feature extraction strategy, which helps capture multi-scale features of targets, enhancing the contrast between targets and the background and thereby improving the detection accuracy of field fruits. Simultaneously, the PPA module employs hierarchical feature fusion and attention mechanisms to maintain and enhance the representational capability of targets, ensuring that key information is preserved during multiple down-sampling processes and reducing feature loss caused by repeated down-sampling. The specific structure is shown in Figure 4.

As shown in the figure above, the PPA module consists of two parts: multi-branch fusion (Patch-Aware and Feature Selection, represented by the blue and yellow background sections) and an attention mechanism (represented by the purple background section). The distinction between local branches and global branches is achieved by controlling the patch-size parameter, computing the attention matrix between non-overlapping patches, and enabling the extraction and interaction of local and global features.

Patch-Aware module (highlighted in cyan in the figure): Firstly, we employ computationally efficient operations (including unfolding and reshaping) to partition F′ into a set of spatially continuous image patches (p × p, H′/p, W′/p, C′). Subsequently, average processing is performed along the channel dimension to obtain features of dimension (p × p, H′/p, W′/p), which are then linearly computed through the feed forward network (FFN). Next, an activation function is applied to the linearly computed features to obtain a probability distribution in the spatial dimension, based on which their weights are adjusted.

Feature selection (highlighted in yellow in the diagram): In the weighted results, we employ a feature selection method to choose task-relevant features from tokens and channels. Firstly, the weighted results are tokens, and each token is re-weighted based on its relevance to the task-embedding vector, thereby effectively simulating the token selection process (i.e., Token Selection). Subsequently, a linear transformation is applied to each token to achieve channel selection, followed by reshaping and interpolation operations (i.e., Channel Selection). Finally, the features F′_local, F′_global, and F′_conv are generated, and their sum yields the feature vector F′.

Feature fusion and attention (highlighted in purple in the diagram): After completing feature extraction through multi-branch feature extraction, we employ an attention mechanism for adaptive feature enhancement. This attention module consists of efficient channel attention and spatial attention components. The feature vector F′ obtained after feature extraction sequentially undergoes one-dimensional channel attention (M_C = 1 × 1 × C′) and two-dimensional spatial attention (M_S = H′ × W′ × 1) processing. Through element-wise multiplication and subsequent activation functions (ReLu), batch normalization (BN), and other operations, the output F″ of the PPA module is obtained.

**Figure 5:**
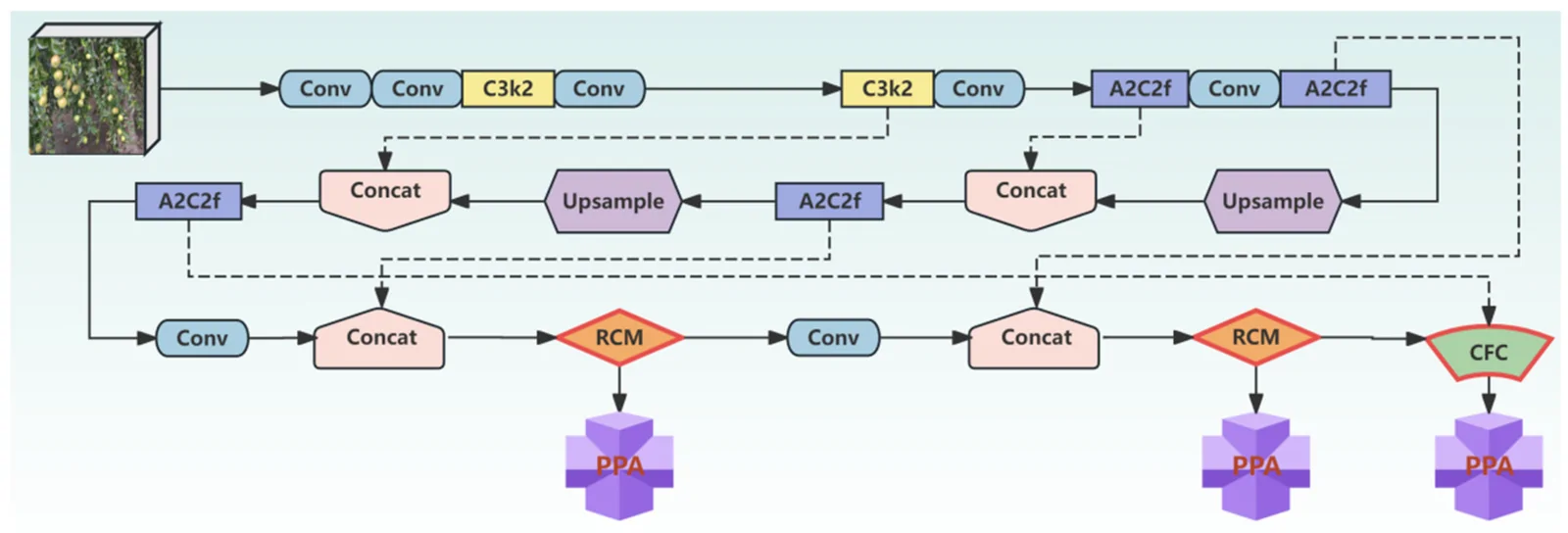
The architecture of the RCPNet.

In conclusion, a schematic diagram illustrating the architecture of the modified network is presented in Figure 5.

In addition, the baseline network introduces a Residual Efficient Layer Aggregation Network (R-ELAN), which optimizes gradient flow through residual design and scaling techniques, and designs feature aggregation methods to enhance model optimization efficiency. This architecture enhances feature aggregation efficiency and alleviates optimization instability inherent in ELAN. Its core innovation lies in a bottleneck-based (light-gray section) feature aggregation mechanism, which reduces channel dimensions and simplifies computational pathways. This design substantially reduces computational overhead and memory footprint while maintaining effective feature fusion.

In near-color scenarios, standard multi-branch attention can suffer from feature homogenization. PPA employs a parallel, patch-wise self-attention strategy that forces the detection head to compare local color features across disjointed image patches. This significantly enhances sensitivity to subtle color transitions (e.g., between green and early-yellow stages) that are critical for selective harvesting.

### 2.3. Experiment Evaluation Index

In order to better quantitatively analyze the performance of the model, this paper uses several commonly used indicators to evaluate neural networks: Precision (P), Recall (R), mean Average Precision (mAP), F1-score, parameters, floating point operations (FLOPs), and model size.

P represents the proportion of correctly identified positive instances among all instances predicted as positive. Conversely, R quantifies the proportion of actual positive instances correctly identified by the model. These metrics are formally defined in Equations (7) and (8), respectively:

(7) $$Recall=\frac{TP}{TP+FN}$$

(8) $$Precision=\frac{TP}{TP+FP}$$

where TP denotes the number of true positives (correctly predicted positive instances), FP represents the number of false positives (incorrectly predicted positive instances), and FN signifies the number of false negatives (actual positives incorrectly predicted as negative).

P and R are typically inversely correlated. Consequently, neither metric alone comprehensively assesses model quality. Therefore, the experiments utilize the F1-score (Equation (9)), defined as the harmonic mean of P and R, to provide a balanced evaluation incorporating both precision and recall.

(9) $$F1=\frac{2\times Precision\times Recall}{Precision+Recall}$$

Average Precision (AP) is defined as the area under the Precision–Recall curve. A higher AP value indicates superior model performance. The mAP@0.5 refers to the mean Average Precision computed at a single IoU threshold of 0.50. mAP@0.5:0.95 refers to the mean Average Precision averaged over IoU thresholds from 0.50 to 0.95 with a step size of 0.05. This metric is formally expressed in Equations (10) and (11).

(10) $$AP=\int_{0}^{1}P(r)dr$$

(11) $$mAp=\frac{\sum_{i=1}^{c}{AP}_{i}\;}{n}$$

where c is the category number, and i is the order number.

Model size refers to the file size of the trained network’s parameter weights. The size of this weight file is primarily determined by the number of learnable parameters in the network architecture. Optimizing the network structure can effectively reduce model size while maintaining detection accuracy. Smaller models are consequently more suitable for deployment on mobile and edge devices. FLOPs serve as a key metric for assessing model computational complexity. A lower FLOP count indicates a less complex model, which translates to reduced computational resource requirements and faster inference times.

### 2.4. Design Rationale and Module Complementarity

The three modules form a sequential pipeline targeting distinct yet interdependent challenges. RCM restores spatial structure in occluded regions through axial self-calibration, but does not separate fruit features from occlusion noise. CFCG then applies a gating mechanism to adaptively calibrate the restored features—enhancing clean foreground signals while suppressing corrupted or ambiguous ones. On these refined features, PPA amplifies subtle color differences between maturity stages, addressing the near-color discrimination problem that the previous two occlusion-oriented modules cannot solve. Together, the pipeline progresses from context restoration to feature denoising and finally to fine-grained color contrast, with each stage resolving the limitation left by its predecessor.

## 3. Experimental Design and Results Analysis

### 3.1. Experimental Platform and Parameter Settings

Model training was conducted on a Lenovo R9000P (Lenovo Group Limited, Beijing, China) laptop equipped with an AMD Ryzen R7-5800H processor (3.2 GHz; AMD, Silicon Valley, CA, USA), 16 GB RAM, and an NVIDIA GeForce RTX3070 GPU (8 GB VRAM; NVIDIA, Santa Clara, CA, USA). The operating system was Windows 10. GPU acceleration was implemented using CUDA 11.3 and cuDNN 8.2.1 libraries. The PyTorch 1.11.0 framework was employed with Python 3.9.1.

**Figure 6:**
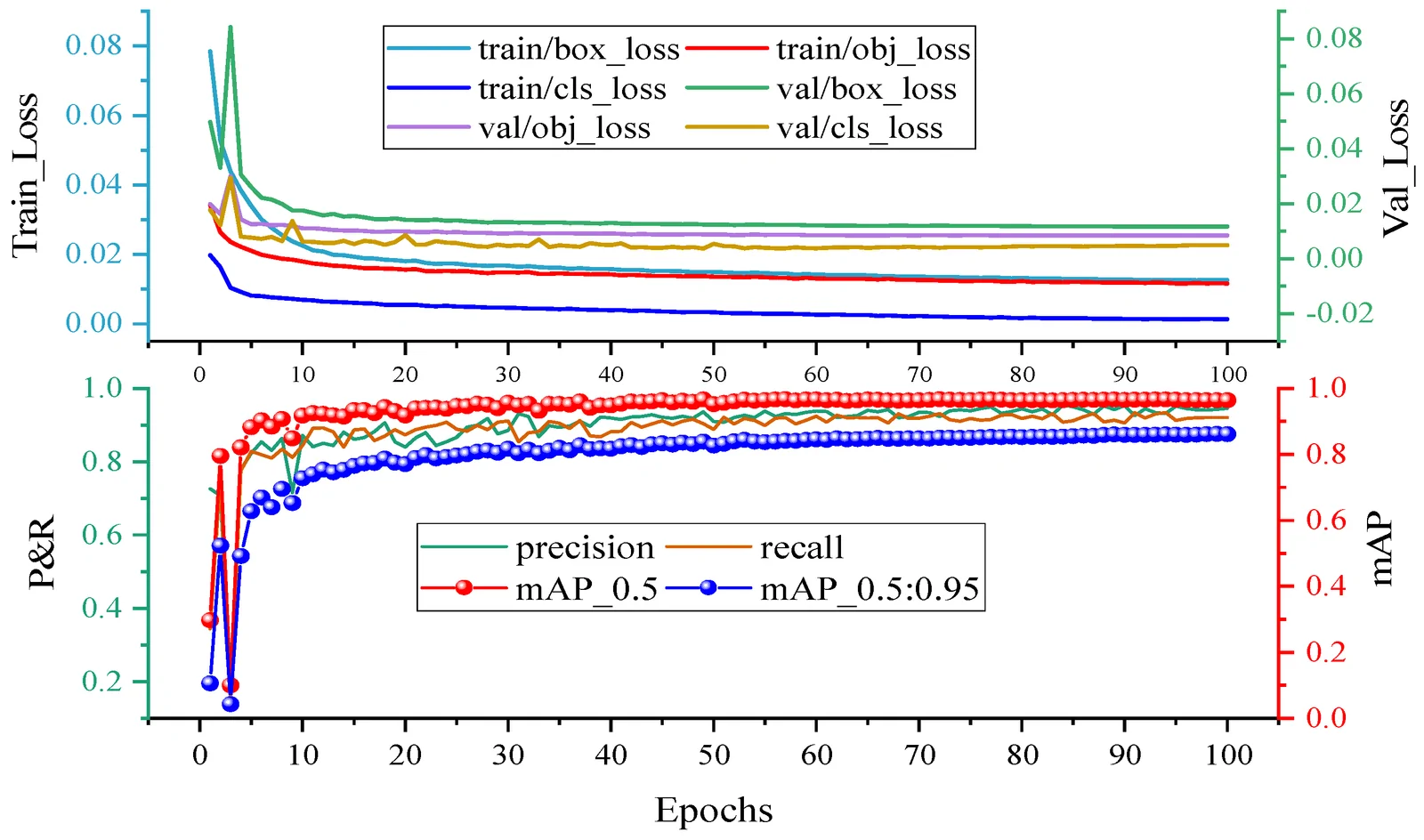
The results of model pre-training.

The network utilized weights pre-trained on the MS COCO dataset via the YOLOv12 architecture. For input images of size 640 × 640 pixels and featuring three color channels, YOLOv12n has a computational cost of 6.0 GFLOPs (floating-point operations per second), comprising 497 layers and 2.52 million parameters. Optimization employed stochastic gradient descent (SGD) with a momentum factor of 0.937 and weight decay of 0.0005. The initial learning rate was set to 0.01, governed by a cosine annealing decay strategy. The detection confidence threshold was set to 0.001 during evaluation, and non-maximum suppression was applied with an IoU threshold of 0.7. All experiments were run with a fixed random seed of 42 for Python, NumPy, and PyTorch. We also repeated the main experiments with three different seeds (42, 123, 2024) to obtain the mean ± std, reported in the result tables. Training utilized a batch size of 32 with four data-loading workers. In this paper, each group of experiments was conducted using the same parameters, with epochs set to 100. The variation in loss during the experiments is shown in Figure 6.

As shown in the graph, the loss value decreases rapidly in the early stages of training and tends to stabilize around 50 epochs, achieving complete convergence without underfitting or overfitting. Notably, this section distinguishes between the initial convergence diagnosis (where only the baseline model undergoes 100 training cycles) and the formal training protocol (where all models complete 50 training cycles).

### 3.2. Image Dehazing and Model Pre-Experiment

#### 3.2.1. Image Dehazing and Data Balancing Experiments

**Figure 7:**
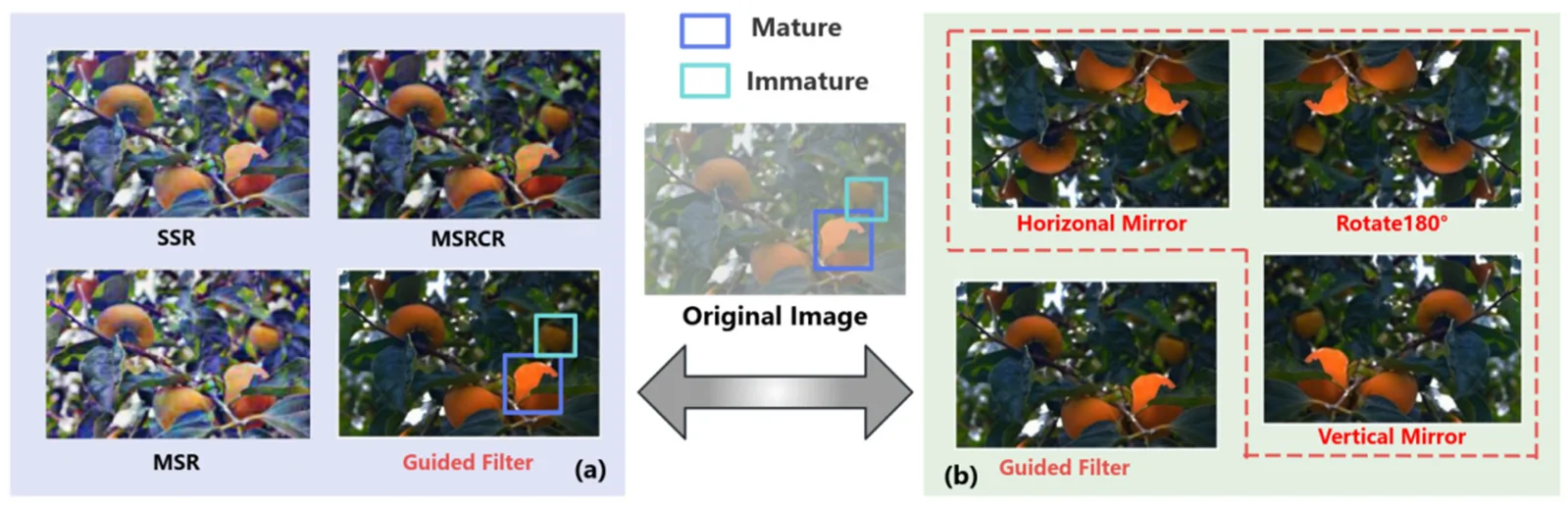
Image dehazing and data balancing. (**a**) Defogging; (**b**) data balancing.

Within sweet persimmon orchards, elevated temperatures and poor ventilation induced haze and blurring effects in partial images, resulting in quality degradation. Furthermore, the substantially higher proportion of mature fruits compared to immature fruits during the harvest period led to imbalanced data distribution, potentially compromising experimental outcomes. To mitigate these issues, this study implemented guided image filtering for haze removal during the preprocessing phase and performed data augmentation on immature fruit images (including horizontal flipping, vertical flipping, and 180° rotation), as detailed in Figure 7.

As illustrated in Figure 7a, four dehazing approaches were implemented: three Retinex-based filtering methods—Single-Scale Retinex (SSR), Multi-Scale Retinex (MSR), and Multi-Scale Retinex with Color Restoration (MSRCR)—alongside Guided Filtering (GF). Empirical results demonstrated that the guided filtering approach achieved superior dehazing performance. A total of 369 images exhibiting blurring effects and immature fruits were selected for data augmentation and balancing (red dashed border). Following enhancement, the ratio of fruit quantities was adjusted from 8:1 to 3:1, with the final dataset expanded to 8701 images (7570 original + 1131 augmented). To prevent any leakage of augmented samples into the evaluation, all data augmentation was performed offline and exclusively on the training subset after the training split. The validation and test sets consist solely of original, unmodified images from the base 7570 pool. No augmented image was ever used for evaluation.

All metrics are mAP@0.5 on the test set, mean ± std over three runs. The mature/immature AP gap shows no significant difference across configurations (*p* > 0.05, paired *t*-test). Complete statistical details are presented in Table 2.

As evidenced in Table 2, the data augmentation contributes +3.1% mAP, while dehazing alone yields a modest +1.8% mAP, and the combined gain of +4.5% mAP is largely additive. Crucially, under all configurations, the AP gap between mature and immature fruits remains statistically indistinguishable (*p* > 0.05), confirming that dehazing does not distort the inter-maturity discriminability.

#### 3.2.2. K-Fold Cross-Validation

Before conducting the main experiments, we first evaluated the stability of the baseline network using K-fold cross-validation. The training set was randomly partitioned into K = 3 equal folds while preserving the class distribution via stratified sampling. For each fold, the baseline network was trained on K-1 folds and validated on the remaining fold. This process was repeated three times, and the performance was reported as mean ± standard deviation.

As shown in Table 3, the baseline network achieved a mean mAP@0.5 of 93.6% (±0.1%) across the three folds. The above analysis shows that the small standard deviations point to stable performance for the baseline model, regardless of how the training and validation data are split, and that this stability is statistically significant (*p* < 0.05).

### 3.3. Algorithm Improvement Experiment

#### 3.3.1. Boosting Contextual Extraction Through Rectangular Self-Calibration Module

Addressing the challenge of intensive mutual occlusion inherent in the clustered growth patterns of sweet persimmons within complex agricultural environments, conventional object detection algorithms demonstrated limited efficacy in handling dense occlusions. Axial contextual information was leveraged to enable the model to infer features within occluded regions, thereby significantly mitigating both false negatives and false positives. Through numerous experimental validations, the A2C2f module at layer 17 and the C3k2 module at layer 20 were strategically replaced with an RCM to notably enhance the model’s capacity for contextual information extraction. Meanwhile, this study used modules targeting context extraction capabilities, such as the Lightweight Adaptive Extraction (LAE), Poly Kernel Inception Network (PKI), Spatially Adaptive Feature Modulation (SAFM), Contextual Transformer (CoT) and Adaptive Fine-Grained Channel Attention (FCA) modules, for comparative experiments to validate the effectiveness of the improved method, with specific data presented in Table 4.

**Figure 8:**
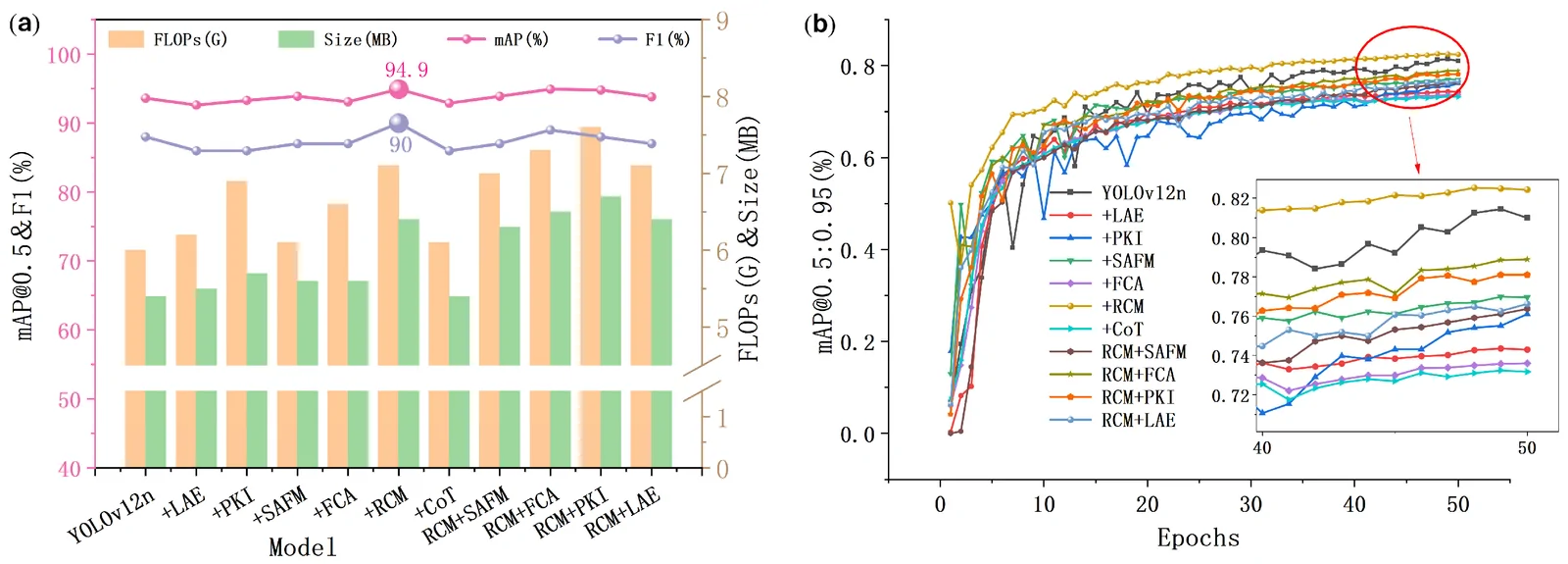
Experimental results of contextual information perception and acquisition. (**a**) Accuracy and complexity metrics; (**b**) mean accuracy of different schemes.

From the data in the above table, it can be observed that this experiment compared and analyzed six modules specifically designed to enhance the perception and extraction of contextual information. In terms of local contextual features, both the LAE [26] and SAFM [27] modules demonstrated adaptive focusing capabilities. However, the LAE module exhibited a restricted perceptual range, and its lightweight design incurred a cost in precision. In contrast, the SAFM module yielded a 0.3% performance improvement by adaptively adjusting feature maps at each spatial position, effectively suppressing irrelevant background elements. Regarding multi-scale and refined contextual features, the PKI [28] and FCA [29] modules offered specific advantages, although both demonstrated limited capacity for modeling long-range dependencies. To address this limitation, the CoT module employed a self-attention mechanism to capture long-range contextual dependencies; nevertheless, it exhibited reduced sensitivity to positional information and local features. The RCM module captured axial global context along the horizontal and vertical dimensions, enabling spatial feature reconstruction and pyramid context extraction. Experimental results demonstrated that the RCM significantly enhanced performance, increasing mAP@0.5, mAP@0.5:0.95, and F1-scores by 1.3%, 1.1%, and 2.0%, respectively. Corresponding visualizations are presented in Figure 8.

As illustrated in Figure 8a, the incorporation of the RCM alone yielded optimal performance, achieving a mean Average Precision (mAP) of 94.9% and an F1-score of 90.0%. These values represent increases of 1.3 and 1.1 percentage points, respectively, compared to the baseline. However, a modest rise in model complexity was observed. This increase was primarily attributed to the dual-path contextual information extraction structure of the RCM, which contributed to higher computational costs. Furthermore, Figure 8b demonstrates that among the eleven evaluated network architectures, the “+RCM” configuration achieved the highest (orange solid line) mAP@0.5:0.95, indicating that this modification provided the most significant performance improvement. The RCM mitigated occlusion effects by enhancing contextual awareness and information capture. This capability compensated for data loss in blocked zones, and enabled robust inference about occluded content through the exploitation of multi-dimensional features.

#### 3.3.2. The Influence of the Context Feature Calibration Gating Module

Adverse factors within the image, such as illumination variations, shadows, and specular reflections, often led to blurring or even loss of local features. Therefore, to address this limitation and further enhance the model’s robustness against occlusions, we incorporated the CFCG module to perform context calibration, building upon the previously introduced RCM (R-Net) dedicated to augmented context extraction. The CFCG module leveraged global features to interpret and reason about these locally ambiguous features. This process enhanced the disambiguation of easily confusable targets and improved the stability and robustness of the model. Conversely, the Convolution and Attention Fusion Module (CAFM) first harnessed the local feature extraction capability of convolutional operations; it then compensated for global dependencies by employing a self-attention mechanism. An alternative approach employed axial attention to more effectively capture global contextual information, thereby enhancing the extraction of multi-scale and multi-range features, such as the Multi-scale Cross-axis Attention (MCA) module. Consequently, the CAFM and MCA modules were designated as reference modules in this experiment to validate the effectiveness of the proposed context calibration method. The specific experimental data are shown in Table 5.

**Figure 9:**
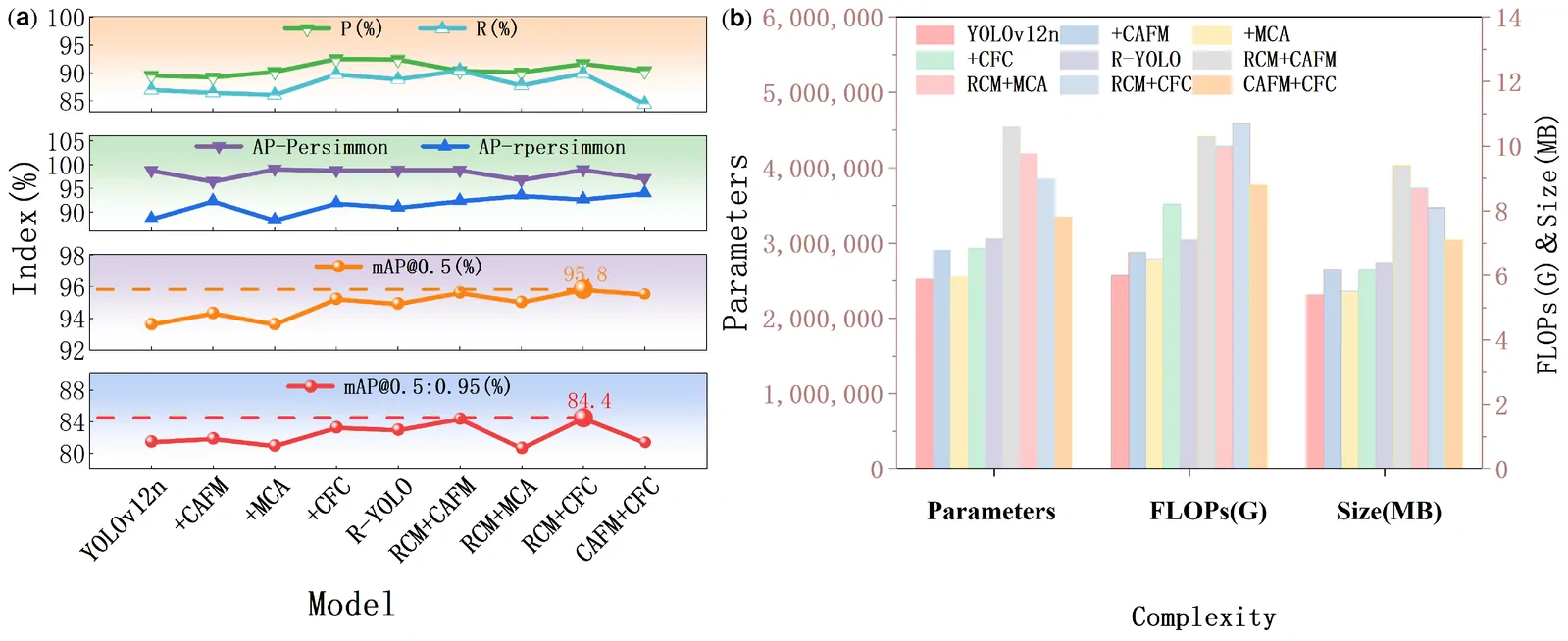
The trend of index change in the contextual calibration experiment. (**a**) Variation curve of accuracy index; (**b**) variation curve of complexity index.

Analysis of Table 5 revealed that the mAP value remained unchanged under the MCA [30] scheme. While the parallel axial attention mechanism enhanced image quality, it failed to overcome the limitations posed by ambiguous local features. An alternative approach involved calibrating contextual features. Both the CAFM and CFCG modules served this purpose, albeit through distinct mechanisms: the CAFM [31] compensated global features using local features, whereas the CFCG module employed global features to interpret local features. The data indicated that the CAFM and CFCG modules increased the mAP by 0.7 and 1.6 percentage points, respectively. Correspondingly, mAP@0.5:0.95 rose by 0.4 and 1.8 percentage points. These results demonstrate the superior efficacy of the CFCG module in boosting model precision. Furthermore, integration of the RCM, designed to augment context extraction capability, led to additional gains, elevating mAP and mAP@0.5:0.95 to 95.8% and 84.4%, respectively. This collectively established the RCM + CFCG (named RC-Net) model as delivering the most significant improvement. Visualizations of these outcomes are presented in Figure 9.

As derived from Figure 9a, the RC-Net combination achieved the highest mAP and mAP@0.5:0.95 values and, more importantly, attained a peak recognition accuracy of 93.9% for immature fruits. Figure 9b indicates that the growth rates of parameter count and model size for RC-Net followed identical ranking trends, but its FLOPs reached the maximum value. This outcome is most plausibly explained by the core mechanism of the CFCG module: the high computational cost required for global feature parsing and its intensive fusion mechanism.

#### 3.3.3. Effects of Multi-Branch Structure on Recognition of Similar-Color Fruits

Addressing the challenge posed by the similar coloration of sweet persimmon at mature and immature stages, which hindered reliable visual distinction, it was necessary to enhance their differentiation. The PPA module, which incorporated a self-attention mechanism, offered advantages in this regard. This architecture employed a multi-branch strategy, which effectively enhanced the contrast between the target fruits and the background. Furthermore, the integration of hierarchical feature fusion technology served to mitigate feature degradation associated with repetitive downsampling operations, particularly critical for distinguishing fruits exhibiting subtle color differences.

In addition, there were various methods to enhance feature extraction and interaction by utilizing multi-branch architectures, such as the repeated stacking of the Reparameterized Convolution based on channel Shuffle (RCS) modules and the three-branch interaction structure of the Triplet Attention (TA) module. Consequently, to conduct a comparative analysis, this study individually incorporated the RCS [32] module, the TA module, and the lightweight Dyhead (DY) detection head—an improvement based on dynamic convolution released in the same year—into the baseline RC-Net framework. The detailed comparative results are summarized in Table 6.

**Figure 10:**
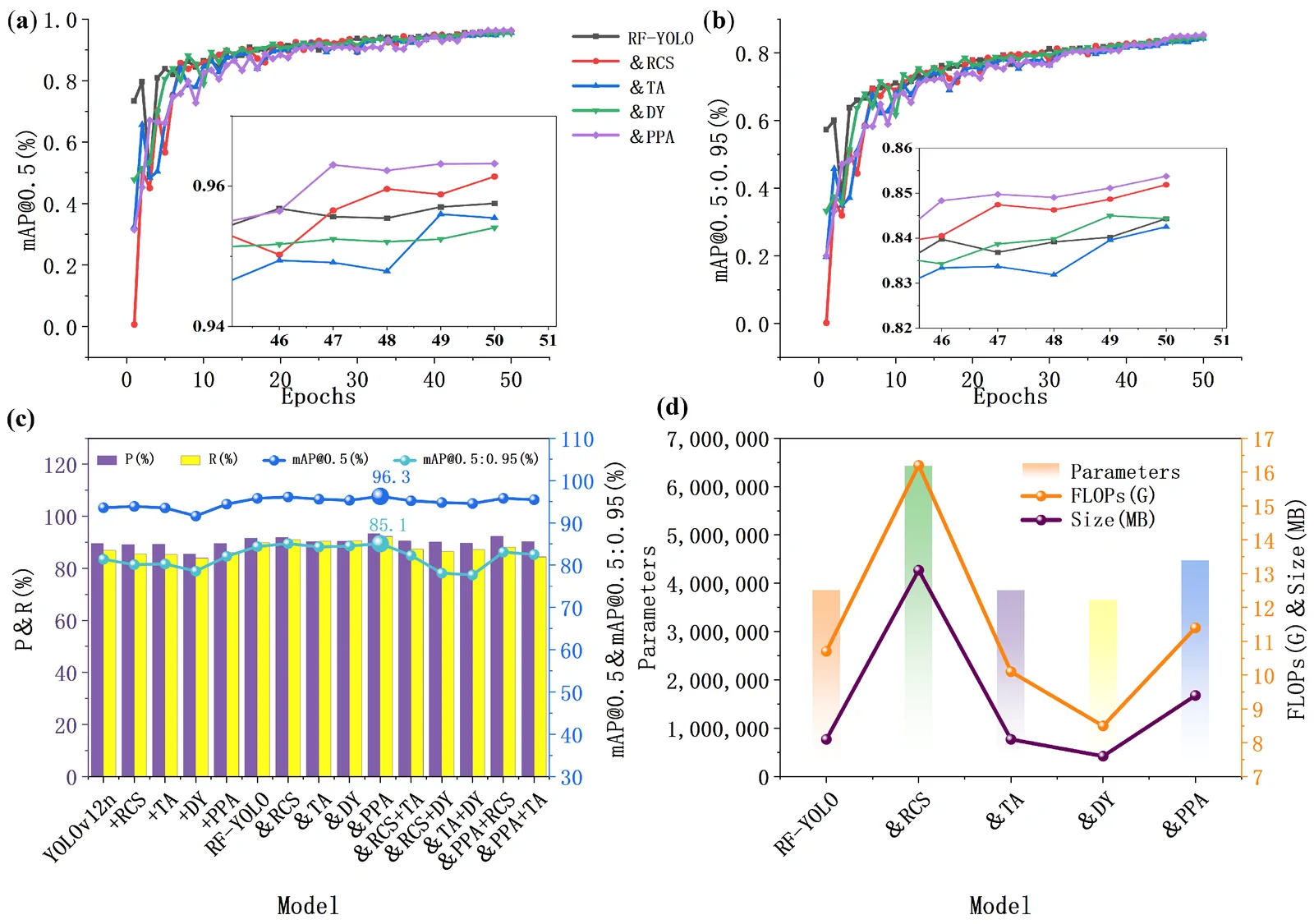
The advantages of the PPA detection head. (**a**,**b**): Comparison of accuracy indicators for four individual methods; (**c**): Utilizing the enhanced RC-Net as the new baseline network, and applying the combinations of the four methods. (**d**): Comparison of complexity indicators for the four individual methods.

Based on the data presented in the table, the analysis first demonstrated that, using the baseline network, the implementation of the four individual improvement strategies yielded respective increases in mAP of 0.3%, −0.1%, −2.0%, and 0.8%. Consequently, the +PPA scheme emerged as the most effective single modification. Subsequently, employing the enhanced RC-Net as the new baseline network, further tests were conducted incorporating the four methods both individually and in combination. These experiments revealed that the &PPA scheme, which integrates the attention mechanism within the detection head, delivered the optimal performance. This approach achieved peak mAP and mAP@0.5:0.95 values of 96.3% and 85.1%, respectively. The scheme of the RCS module likewise contributed to accuracy enhancement. Its recurrent stacking architecture promoted feature reuse and strengthened inter-channel information flow within adjacent feature layers. By employing a multi-branch topological structure to extract richer feature representations, this approach achieved mAP and mAP@0.5:0.95 values of 96.1% and 85.1%, respectively. Nevertheless, the recurrent stacking resulted in increases in model parameters, FLOPs, and size, thereby elevating computational overhead. As a comparative scheme, the Dyhead [33] detection head exhibited advantages in dynamic attention and computational lightness. However, its reliance on deformable convolution introduced excessive sensitivity to positional shifts. This limitation resulted in insufficient capability for resolving boundaries between proximal-color features and reconstructing fragmented features, leading to significant precision degradation. However, the triple-branch structure employing the TA [34] attention mechanism kept model complexity unchanged, but it did not yield an improvement in precision. This phenomenon may be attributed to the concurrent enhancement of proximal-color features as salient features, while the discriminability between these features remained largely unaltered, resulting in minimal fluctuation in the final precision metrics. Furthermore, the various combinations of these four improvement schemes did not yield satisfactory results in terms of both accuracy and complexity metrics. The visualization results are presented in Figure 10.

Magnified views in Figure 10a,b revealed the superior performance of the PPA scheme (denoted by the purple solid line) across both mAP and mAP@0.5:0.95 metrics compared to the other three improvement strategies. Figure 10c further confirms the advantage of this scheme under diverse combinatorial scenarios (represented by blue and cyan spheres). Additionally, Figure 10d demonstrates that integrating the self-attention-equipped PPA detection head into the RC-Net baseline network yielded a further enhancement in precision, accompanied by only a marginal increase in model complexity (reflected by its position as the second smallest increment).

#### 3.3.4. Hyperparameter Sensitivity Analysis

To assess the robustness of the proposed design, we evaluated the sensitivity of two essential hyperparameters: the gating temperature τ in CFCG and the patch size s in PPA. All experiments were conducted on the sweet persimmon validation set using the optimal model configuration reported in Section 3.3.3.

(1) The parameter τ controls the softness of the gating mechanism during context feature calibration. A smaller τ makes the gate harder (closer to binary), while a larger τ softens the decision. We tested τ ∈ {0.5, 1.0, 1.5} with the default patch size s = 8. (2) The patch size s defines the spatial resolution at which patch-wise self-attention is performed. A smaller s captures finer details but increases computational cost, while a larger s may overlook subtle color differences. We examined s ∈ {4, 8, 16} with the default gating temperature τ = 1.0. The results are summarized in Table 7.

As shown in Table 7, the model performance is remarkably stable across a wide range of both parameters. For τ, the mAP varies by only 0.2% between the best (τ = 1.0) and the worst (τ = 0.5), and the mAP@0.5:0.95 remains within a 1.1% band. Similarly, for patch size s, the results are nearly identical for s = 4 and 8, with a slight drop when the patch becomes too large (s = 16). These results confirm that the chosen default settings (τ = 1.0, s = 8) are close to optimal and that the model is not overly sensitive to either hyperparameter. This robustness further supports the practical reliability of the proposed architecture.

### 3.4. Ablation Experiment

The ablation experiment aligns with established scientific principles of causal analysis and is crucial for transparency in model design and the reproducibility of research claims. Therefore, ablation experiments were conducted to validate the effectiveness of each improvement strategy. The results are shown in Table 8.

As shown in Table 8, while individually integrating RCM, PPA, and CFCG yielded mAP@0.5 gains of +1.3%, +1.0%, and +1.6%, respectively (a naive linear sum of +3.9%), the complete RCPNet model achieved a +2.7% improvement. This sub-linear additive effect indicates a degree of functional overlap, where the modules partially address the same failure cases (e.g., heavily occluded small fruits). However, it is crucial to note that no single module or partial combination could simultaneously address both challenging scenarios (severe occlusion and color similarity) as effectively as the full RCPNet. Specifically, models equipped with only the highest-gain module (CFCG, +1.6%) still suffered from a 2.5% lower recall on occluded fruits compared to the complete model. Therefore, the complete combination, while exhibiting overlapping gains in mAP, delivers the most balanced and robust detector for the target agricultural deployment.

Analysis of the data presented in rows 1–4 of Table 8 revealed that among the three enhancement strategies, the CFCG module made the most significant contribution, while the PPA detection head contributed the least. This ranking was consistent with the contribution order observed for the combined strategies in rows 5–7. The optimal improvement strategy was as follows: starting from the baseline network, the RCM was employed to capture axial contextual information, thereby enhancing feature perception capabilities within dense environments. Building upon this, the CFCG module was integrated to calibrate this contextual information, thereby enhancing the network’s processing capabilities under conditions of dense occlusion. Finally, a PPA detection head incorporating self-attention was utilized to mitigate interference arising from similar colors of fruits at different maturity stages.

**Figure 11:**
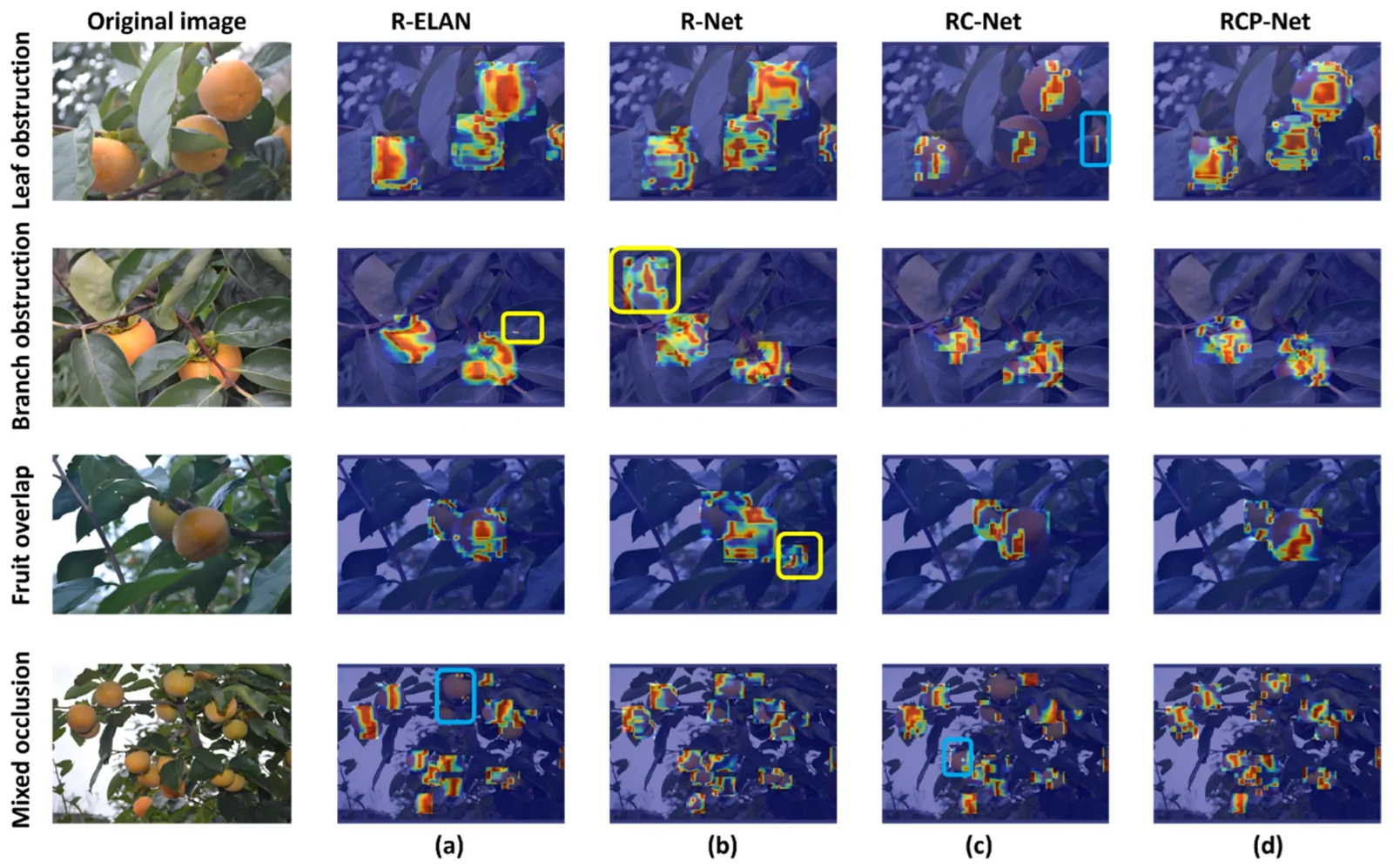
Heatmap of improvement strategies. Yellow rectangle: false detection. Blue rectangle: missed detection. (**a**) R-ELAN; (**b**) R-Net; (**c**) RC-Net; (**d**) RCP-Net.

Under varying occlusion conditions, fruits with similar color characteristics exhibited marked variations in visual features. To qualitatively illustrate the feature attention patterns of each enhancement strategy, EigenGradCAM visualizations were implemented with confidence threshold and ratio parameters set to 0.2 and 0.02, respectively, while maintaining identical configurations for all other parameters. Note that the confidence threshold of 0.2 and ratio of 0.02 were intentionally set lower than typical detection thresholds, in order to capture weak but diagnostically relevant attention signals for our fine-grained maturity discrimination task. The heatmaps for each stage are shown in Figure 11.

As evidenced in Figure 11a, the baseline network exhibited susceptibility to missed detections and false positives under mixed occlusion conditions. Following the integration of the RCM module for enhanced contextual feature capture, attention weights over occluded regions increased substantially (Figure 11b), albeit at the cost of introducing additional false positives. Subsequent calibration via the CFCG module effectively suppressed false detections (Figure 11c), but concurrently reduced the attention coverage on fruits with similar color characteristics—a phenomenon potentially attributable to diminished feature discriminability in near-identical color spaces. Ultimately, incorporation of the self-attention-equipped PPA detection head significantly augmented feature attention toward occluded, similar-color fruits. Removing any single module caused a significant drop in specific subsets of challenging cases, even if the overall mAP gain appeared partly redundant. Thus, the full ensemble is essential for a robust detector.

**Figure 12:**
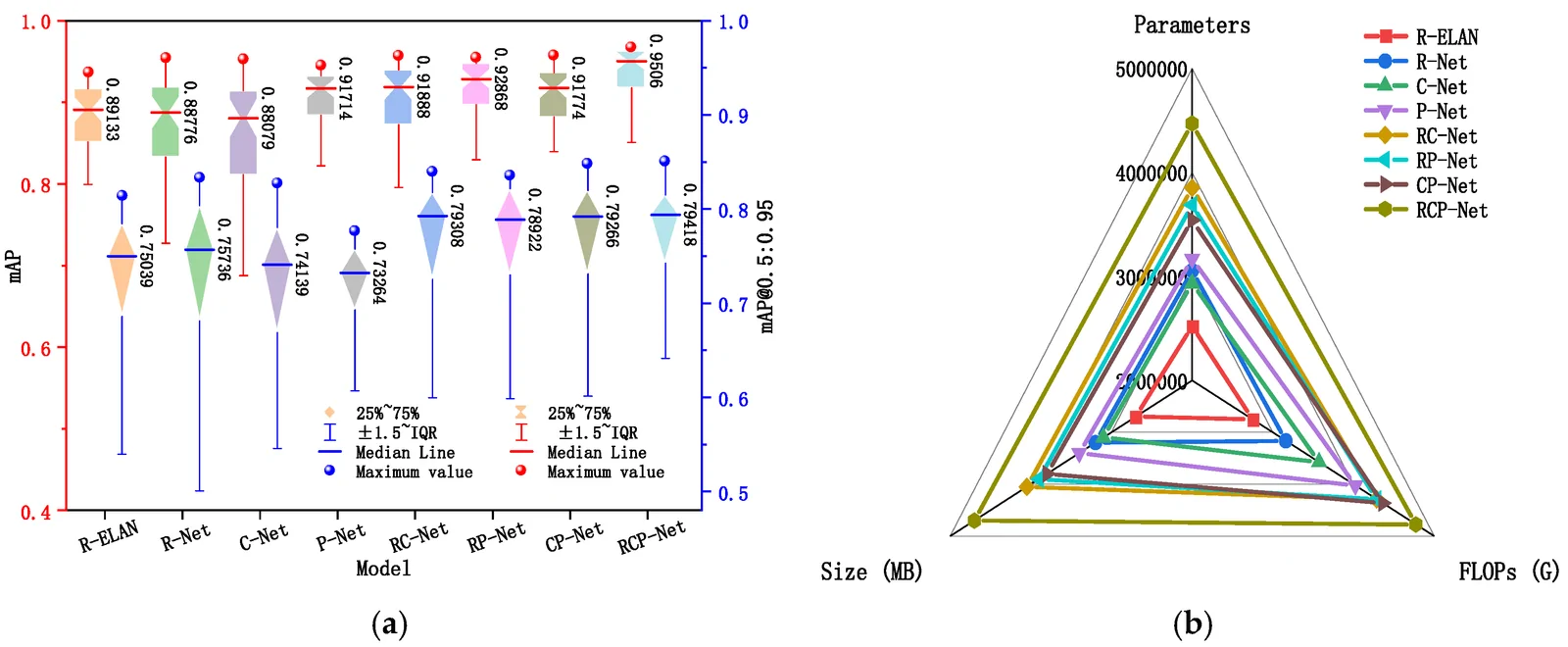
Visualization analysis of ablation experiment results. (**a**) Accuracy comparison: the median line represents the overall precision of the model, while the interquartile range (IQR) quantifies performance stability. The maximum and minimum values of the whiskers reflect the best potential and the lowest limit of the model’s accuracy, respectively. (**b**) Complexity comparison.

The optimized network, RCPNet, demonstrated the best overall performance, achieving final mAP and mAP@0.5:0.95 scores of 96.3% and 85.1%, respectively. These results represent increases of 2.7 and 3.7 percentage points. Furthermore, the data from Table 6 were visualized; the corresponding results are shown in Figure 12.

Analysis of the dual-axis box plot in Figure 12a revealed that the RCPNet network achieved the highest median (red and blue solid lines) and maximum values (red and blue spheres) for both mAP and mAP@0.5:0.95 metrics, indicating superior overall precision. The most compact IQR box further attested to its stable performance across evaluations. Additionally, the elevated lower whisker position demonstrated enhanced robustness, reflecting a higher precision baseline. Concurrently, the radar chart in Figure 12b revealed a progressive increase in model complexity and computational costs associated with the refined algorithm. The extra FLOPs predominantly activated on images with simultaneous severe occlusion and color ambiguity—cases where partial models failed. Removing any module to save complexity led to unacceptable recall drops (>5%) on these critical edge cases, which were the primary failure modes for harvesting robots. Therefore, future research should prioritize corresponding lightweight strategies to address this limitation.

Guided by this analysis, we now firmly position RCPNet as a carefully engineered system that achieves the optimal Pareto-frontier between robustness and complexity for in-canopy fruit detection. For example, RCPNet incorporates only the RCM into CP-Net, incurring an overhead of just 0.2 M parameters, 0.5 G FLOPs and 0.4 MB of model volume, while boosting precision and recall by 2.6 and 1.7 percentage points, respectively. Complete model (2.7% gain) does so with only a marginal increase in parameters (1.22 M) and model size (2.6 MB), achieving a superior gain-per-cost ratio compared to any single-module variant. This practical efficiency is the central engineering contribution.

### 3.5. Comparative Experiment of Mainstream Algorithms

To further validate algorithm efficacy, RCPNet was compared with mainstream single-stage detectors, comprising lightweight variants (SSD, RT-DETR, HCMS, LUDY, YOLOv5n, YOLOv8n, YOLOv10n, YOLOv11n, YOLOv12n, YOLOv13n) and the larger-scale counterpart YOLOv12s from the same series. All values are reported as mean ± standard deviation over three independent training runs with different random seeds. An asterisk (*) indicates that RCPNet is significantly better than the second-best model according to a paired *t*-test (*p* < 0.05). Quantitative comparisons are presented in Table 9.

**Figure 13:**
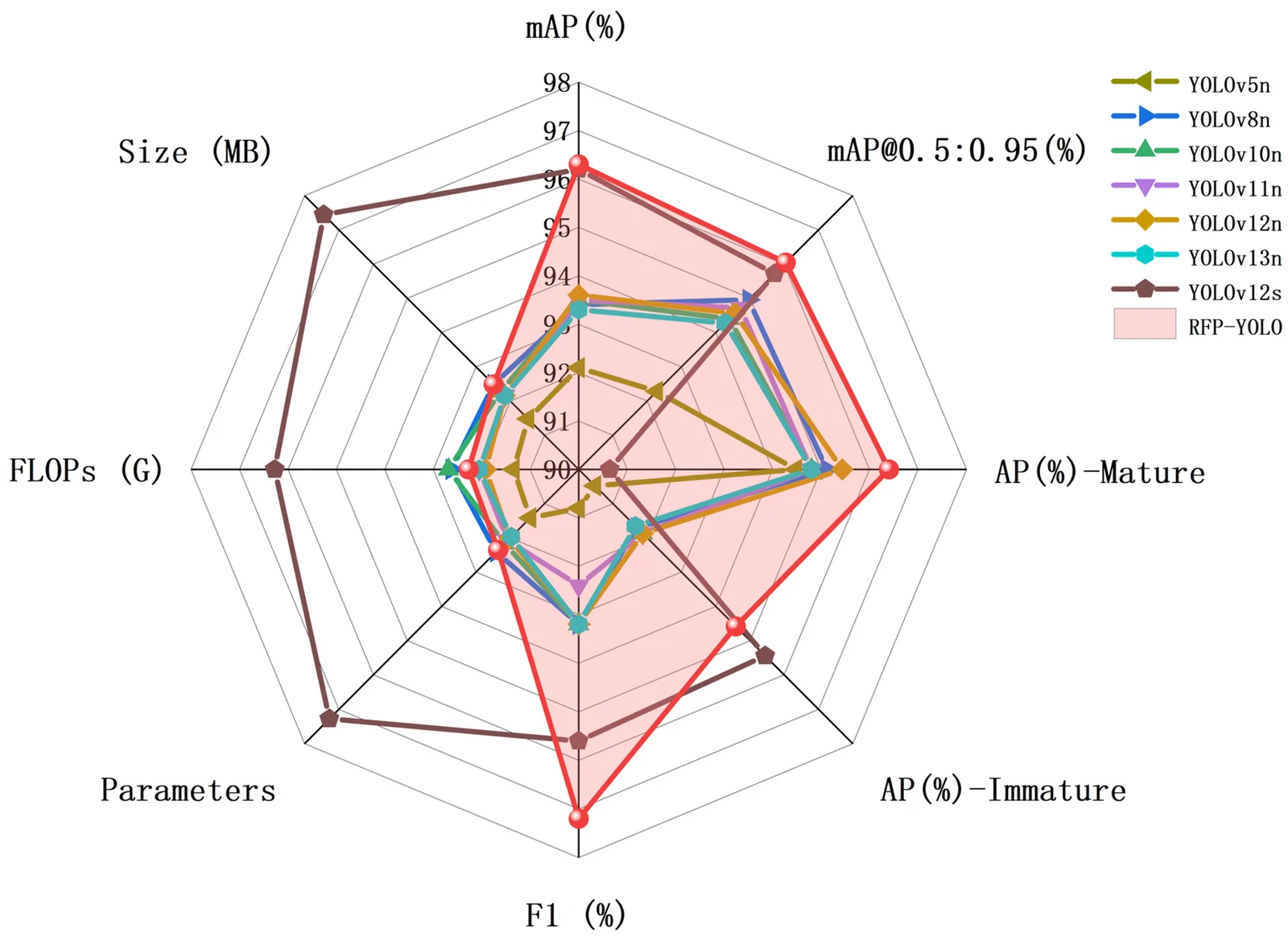
Comparative analysis with mainstream networks. The left side represents the complexity metrics, and the right side represents the accuracy metrics.

Analysis of Table 9 revealed that the RCPNet outperformed both non-YOLO methods significantly. Specifically, compared to RT-DETR-R18, our model achieved a +2.7% higher mAP@0.5 while requiring only 22.2% of its FLOPs and 18.4% of its parameters, underscoring the effectiveness of our agricultural-specific design over general-purpose real-time Transformers. Moreover, RCPNet consistently achieved higher accuracy metrics than mainstream networks of comparable scale. However, the drawback was that the parameters, FLOPs, and model size were all slightly higher compared to the same-scale (nano-scale) network. Furthermore, its performance met or even exceeded that of the larger-scale YOLOv12s network within the same series on several key measures. However, the detection accuracy for immature fruits was notably lower (by 1.6 percentage points) compared to YOLOv12s. This performance gap stemmed primarily from the superior representational capacity of the larger YOLOv12s model in learning and differentiating features from fruits exhibiting similar color characteristics. A notable advantage, however, was its significantly reduced computational footprint. Specifically, the RCPNet model achieved reductions in parameter count, FLOPs, and model size to only 58.86%, 44.90%, and 56.99% of those of the YOLOv12s network, respectively. The comparison of specific data is shown in Figure 13.

We acknowledge that RCPNet introduced additional computational cost over the baseline. However, this increase did not push the model beyond the deployable complexity envelope. As shown in Table 9, our RCPNet achieved 0.1% higher mAP@0.5 than YOLOv12s, while requiring less parameters (3.74 M vs. 9.10 M) and lower FLOPs (10.8 G vs. X19.6 G). Given that YOLOv12s is itself widely regarded as deployable on agricultural robot edge devices, RCPNet’s hardware requirements are demonstrably within the same feasible regime—despite its benchmark model being the smaller nano version.

To better reflect real-world applicability, we evaluated two additional deployment-oriented metrics: inference latency and GPU memory consumption. The experiments were performed on a single NVIDIA RTX 3070 GPU with a batch size of 1, using the same input resolution as in the detection experiments.

As shown in Table 10, RCPNet incurred an additional latency of only 0.2 ms compared to the baseline, which corresponds to a negligible overhead for object detection. The GPU memory footprint increased from 2.96 G to 2.98 G, still easily accommodated by standard desktop GPUs and high-end embedded platforms. This moderate increase is justified by the significant gains in detection accuracy, especially for heavily occluded and similar-color fruits. These results, combined with the previously reported parameter count and FLOPs, confirm that RCPNet maintains a favorable balance between accuracy and computational efficiency, making it suitable for practical agricultural monitoring and picking scenarios.

### 3.6. Visual Comparison Under Challenging Scenarios

**Figure 14:**
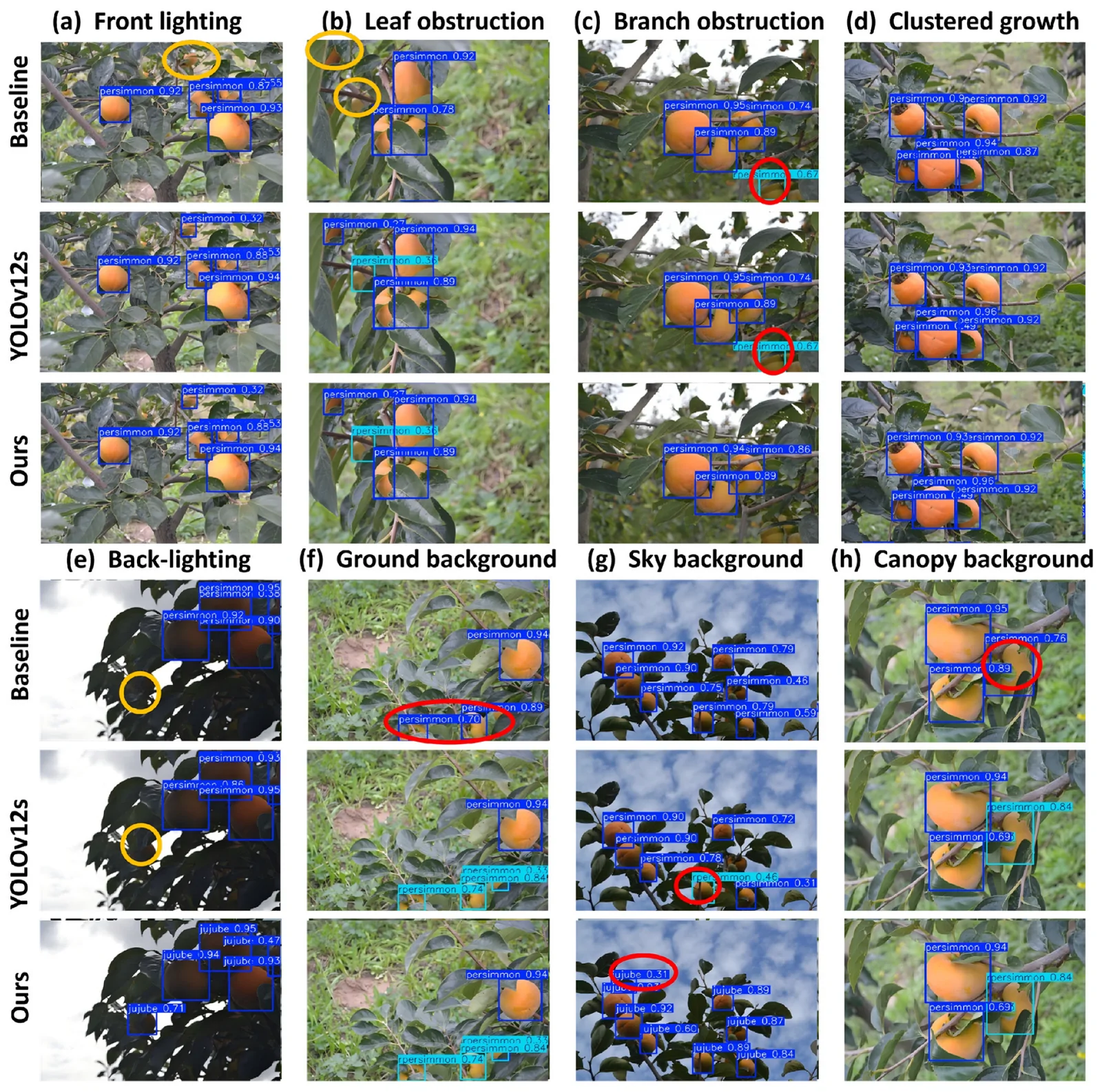
Result image of visual contrast in complex scenarios.

To qualitatively assess the performance of the proposed method, we visually compared RCPNet with baseline and YOLOv12s on a set of representative test images selected to cover eight challenging conditions prevalent in sweet persimmon orchards, with comprehensive results detailed in Figure 14.

In addition to these success cases, we also illustrate the remaining limitations in Figure 14. Specifically, fruits that were almost entirely covered (over 90% occlusion) or those located at the image periphery with severe blur still posed difficulties for RCPNet, as did maturity pairs that were virtually indistinguishable in the RGB color space. These cases suggest that future work could incorporate additional modalities (e.g., near-infrared or depth) to further improve robustness.

Overall, the visual comparisons consistently align with the quantitative results, confirming that RCPNet offers practical advantages for detection in complex orchard environments while also revealing clear directions for further improvement.

## 4. Discussion

This work focused on the performance drop that fruit detection models suffer in orchards when fruits are heavily occluded or share similar colors. The experiments showed that RCPNet, which integrates three dedicated modules (RCM, CFCG, and PPA), achieves higher detection accuracy than the baseline and several mainstream detectors. Its computational overhead remains small. These results confirm that targeted architectural changes can effectively address specific agricultural vision problems.

RCPNet performed consistently on sweet persimmon and flat jujube datasets despite marked differences in shape, color, and canopy. This suggests no overfitting to a single fruit type, consistent with earlier findings that task-specific geometric priors transfer better than purely learned features [35]. With a peak mAP of 96.3% and a modest model size, RCPNet provides a practical accuracy–efficiency balance. Its modular design can serve as a reference for other fruit crops facing similar occlusion and similar-color challenges. (1) The RCM enforces rectangular, axis-aligned context aggregation. Deformable convolution [36] learns free-form offsets, but replacing the RCM with it reduced recall on heavily occluded fruits. Clustered growth causes directional occlusion, and unconstrained offsets overfit background textures when training data are limited. The rectangular prior matches the elongated cluster shape, improving data efficiency and robustness. (2) CFCG uses a per-position gating mechanism for feature calibration. Standard channel attention [37] applies uniform weights across all positions, which cannot separate fruit features from leaf-corrupted ones in occluded scenes. CFCG suppresses ambiguous signals while retaining a clean foreground, acting as a denoising step after the RCM restores spatial structure. (3) PPA employs parallel patch-wise self-attention for near-color separation. Global self-attention [38] can dilute subtle local color contrasts. PPA isolates color information within small patches before aggregation. The consistent gain in immature fruit AP confirms the benefit of amplifying local color contexts.

Several limitations remain. First, under extreme occlusion (over 90% of the area hidden), the RCM has almost no visible context to restore, and CFCG receives too little reliable foreground to calibrate. In such cases, detection often fails entirely. Multi-modal sensing, such as depth or thermal imaging, may provide geometric cues that are independent of visible texture [39]. Second, fruits at the far end of rows or at image boundaries can become very small targets, occupying fewer than 20 pixels in width. The current feature pyramid may not preserve sufficient detail for these instances. Lightweight super-resolution or adaptive zoom strategies could be explored [40]. Third, complex lighting conditions, particularly strong backlight combined with deep shadow, shift color values outside the training distribution. This degrades PPA’s maturity discrimination. Training with physically grounded color augmentations or adopting perceptual color spaces may improve robustness. Fourth, the current model was evaluated on two datasets collected under similar geographic and climatic conditions. Performance on orchards with different tree architectures, pruning practices, or cultivars remains unknown. Domain adaptation or test-time training strategies would be valuable for broader deployment. Finally, although RCPNet shows acceptable GPU latency, no test on embedded hardware was performed. Real-time operation on picking robots requires benchmarking on platforms such as Jetson Orin, along with model pruning and quantization. These engineering steps are essential before practical adoption.

## 5. Conclusions

This paper presented RCPNet, a detection model built for orchard scenarios with dense occlusion and similar-color fruits. The model combines an RCM for directional context capture, a CFCG for adaptive feature denoising, and a PPA head for fine-grained color discrimination. On a self-collected sweet persimmon dataset, RCPNet achieved a peak mAP of 96.3% and mAP@0.5:0.95 of 85.1%, outperforming the baseline and several mainstream detectors with only a small increase in computational complexity. Its generalization was validated on a separate flat jujube dataset, where consistent improvements were also observed. These results demonstrate that RCPNet provides an effective and practical solution for automated fruit recognition in complex field conditions. The modular and task-driven design offers a solid foundation for extending the approach to other fruit crops and agricultural vision tasks.

## Figures and Tables

**Table 1:** Distribution of datasets.

Dataset	Number	Anchor		
		Mature	Immature	Total
Training	6960	16,666	5780	22,446
Validation	870	2092	779	2871
Test	871	2151	665	2816
Total	8701	20,909	7224	28,133

**Table 2:** The results of the data-balancing experiment.

Dataset	Anchors		P (%)	R (%)	AP (%)		mAP@0.5 (%)	F1 (%)
	Mature	Immature			Mature	Immature		
Raw	19,745	2527	84.2	82.4	98.6 ± 0.3	79.7 ± 0.4	89.1 ± 0.2	86
+GF			85.4	86.8	98.5 ± 0.2	82.2 ± 0.3	90.9 ± 0.1	86
+Balanced	20,909	7224	87.8	86.2	98.5 ± 0.3	85.9 ± 0.4	92.2 ± 0.3	87
Balanced & GF			89.5	86.9	98.7 ± 0.2	88.5 ± 0.3	93.6 ± 0.2	88

**Table 3:** Validation results of the K-fold cross-validation.

K-Fold Cross-Validation	P (%)	R (%)	F1 (%)	AP (%)		mAP@0.5 (%)	mAP@0.5:0.95 (%)
				Mature	Immature		
Fold 1	88.2	86.4	88	98.5	88.5	93.5	80.3
Fold 2	90.2	86.0	88	99.0	88.2	93.6	80.7
Fold 3	90.1	87.3	88	98.6	88.8	93.5	80.5
Mean ± Std	89.5 ± 0.6	86.9 ± 0.5	88 ± 0.0	98.7 ± 0.3	88.5 ± 0.4	93.6 ± 0.1	80.5 ± 0.5

**Table 4:** Experimental results of contextual information extraction.

Model	P (%)	R (%)	mAP@0.5 (%)	mAP@0.5:0.95 (%)	F1 (%)	Parameters	FLOPs (G)	Size (MB)
Baseline (R-ELAN)	89.5	86.9	93.6	81.4	88	2,520,054	6.0	5.4
+LAE	88.0	84.8	92.6	74.3	86	2,078,550	6.2	5.5
+PKI	87.3	85.6	93.3	76.1	86	2,694,246	6.9	5.7
+SAFM	89.1	85.5	93.9	77.0	87	2,674,294	6.1	5.6
+FCA	89.7	84.0	93.1	73.6	87	2,615,932	6.6	5.6
+RCM	92.4	88.8	94.9	82.9	90	2,746,278	6.9	5.8
+CoT	90.0	83.2	92.9	73.3	86	2,552,964	6.1	5.4
RCM + SAFM	90.0	84.6	93.9	76.4	87	3,234,742	7.0	6.3
RCM + PKI	89.7	89.4	94.9	78.9	89	3,122,620	7.3	6.5
RCM + FCA	90.4	86.4	94.8	78.1	88	3,200,934	7.6	6.7
RCM + LAE	88.4	86.2	93.8	76.6	87	2,808,406	7.1	6.4

**Table 5:** Experimental results of contextual calibration.

Model	P (%)	R (%)	F1 (%)	AP (%)		mAP@ 0.5 (%)	mAP@ 0.5:0.95 (%)	Parameters	FLOPs (G)	Size (MB)
				Mature	Immature					
R-ELAN	89.5	86.9	88	98.7	88.5	93.6	81.4	2,520,054	6.0	5.4
+CAFM	89.2	86.4	88	96.4	92.2	94.3	81.8	2,902,527	6.7	6.2
+MCA	90.2	86.0	88	99.0	88.2	93.6	80.9	2,550,016	6.5	5.5
+CFCG	92.5	89.7	90	98.7	91.8	95.2	83.2	2,932,054	8.2	6.2
R-Net	92.4	88.8	90	98.8	90.9	94.9	82.9	2,746,278	6.9	5.8
RCM + CAFM	90.3	90.4	90	98.8	92.3	95.6	84.3	4,536,895	10.3	9.4
RCM + MCA	90.1	87.7	90	96.7	93.4	95.0	80.6	4,188,470	10.0	8.7
RCM + CFCG	91.6	89.9	91	97.7	93.9	95.8	84.4	3,128,326	8.6	6.6
CAFM + CFCG	90.3	84.4	90	98.4	92.6	95.5	81.3	3,345,183	8.8	7.1

**Table 6:** Experimental results of the multi-branch structure.

Model	P (%)	R (%)	mAP@0.5 (%)	mAP@0.5:0.95 (%)	Parameters	FLOPs (G)	Size (MB)
R-ELAN	89.5	86.9	93.6	81.4	2,520,054	6.0	5.4
+RCS	89.1	85.5	93.9	80.1	5,127,014	12.6	10.5
+TA	89.3	85.3	93.5	80.3	2,550,306	6.5	5.5
+Dyhead	85.5	83.9	91.6	78.6	2,347,022	5.0	5.1
+PPA	89.5	86.1	94.4	82.1	3,168,730	9.4	6.8
RC-Net	91.6	89.9	95.8	84.4	3,128,326	8.6	6.6
&RCS	91.9	91.0	96.1	85.1	6,429,974	16.2	13.1
&TA	90.3	90.4	95.6	84.3	3,853,266	10.1	8.1
&Dyhead	90.4	90.6	95.3	84.2	3,649,882	8.5	7.6
&PPA	93.3	92.2	96.3	85.1	3,742,954	10.8	8.0
&RCS + TA	90.5	87.3	95.2	82.3	6,430,274	16.2	13.1
&RCS + Dyhead	90.1	86.4	94.8	78.1	6,226,990	14.7	12.7
&TA + Dyhead	89.6	87.2	94.6	77.7	3,650,282	8.6	7.7
&PPA + RCS	92.2	88.1	95.8	83.1	4,471,990	11.4	9.4
&PPA + TA	90.3	84.4	95.5	82.5	7,048,696	17.5	14.5

**Table 7:** Hyperparameter sensitivity results.

Hyperparameter	Value	P (%)	R (%)	mAP@0.5 (%)	mAP@0.5:0.95 (%)	AP (%)		F1 (%)
						Mature	Immature	
τ (gating temp.) default s = 8	0.5	92.8	90.1	96.0 ± 0.3	84.0 ± 0.5	99.0	92.9	92
	1.0	93.3	92.2	96.3 ± 0.2	85.1 ± 0.3	99.0	93.6	93
	1.5	94.2	90.7	96.2 ± 0.2	84.6 ± 0.4	99.0	93.4	92
s (patch size) default τ = 1.0	4	93.9	88.7	96.1 ± 0.2	84.4 ± 0.3	99.1	93.2	91
	8	93.3	92.2	96.3 ± 0.2	85.1 ± 0.3	99.0	93.6	93
	16	92.2	89.4	95.9 ± 0.2	84.1 ± 0.4	99.1	92.7	91

**Table 8:** Results of the Ablation Experiment.

Model	RCM	CFCG	PPA	P (%)	R (%)	mAP@0.5 (%)	mAP@0.5:0.95 (%)	Parameters	FLOPs (G)	Size (MB)
R-ELAN	×	×	×	89.5	86.9	93.6	81.4	2,520,054	6.0	5.4
R-Net	√	×	×	92.4	88.8	94.9 (+1.3)	82.9	2,746,278 (+226,224)	6.9	5.8
C-Net	×	√	×	92.5	89.7	95.2 (+1.6)	83.2	2,932,054 (+412,000)	8.2	6.2
P-Net	×	×	√	89.6	87.2	94.6 (+1.0)	82.7	3,168,730 (+648,676)	9.4	6.8
RC-Net	√	√	×	91.6	89.9	95.8 (+2.2)	84.4	3,128,326 (+608,272)	8.6	6.6
RP-Net	√	×	√	89.9	90.9	95.7 (+2.1)	84.6	3,369,098 (+849,044)	9.9	7.2
CP-Net	×	√	√	90.7	90.5	95.8 (+2.2)	84.9	3,542,586 (+1,022,532)	10.3	7.6
RCPNet	√	√	√	93.3	92.2	96.3 (+2.7)	85.1	3,742,954 (+1,222,900)	10.8	8.0

**Table 9:** Experimental comparison between the improved network and the mainstream network.

Module	mAP@0.5 (%)	mAP@0.5:0.95 (%)	AP (%)		F1 (%)	Parameters	FLOPs (G)	Size (MB)
			Mature	Immature				
SSD	88.6 ± 0.6	—	97.5	79.6	82	26,796,846	35.2	91.1
RT-DETR-18	93.6 ± 0.4	82.4 ± 0.7	98.7	88.6	88	20,321,685	48.6	38.6
HCMS	92.9 ± 0.5	80.2 ± 0.6	98.3	87.5	88	2,995643	8.4	6.4
LUDY	92.5 ± 0.6	79.6 ± 0.8	98.5	86.5	86	2,812,632	9.2	5.6
YOLOv5n	92.1 ± 0.5	75.7 ± 0.6	98.4	85.9	85	1,766,623	4.2	3.7
YOLOv8n	93.4 ± 0.4	82.4 ± 0.5	98.6	88.2	88	3,011,238	8.2	6.2
YOLOv10n	93.5 ± 0.3	81.0 ± 0.5	98.5	88.5	88	2,707,820	8.4	5.7
YOLOv11n	93.5 ± 0.4	81.8 ± 0.8	98.5	88.5	87	2,590,230	6.4	5.4
YOLOv12n	93.6 ± 0.3	81.4 ± 0.5	98.7	88.5	88	2,520,054	6.0	5.4
YOLOv12s	96.2 ± 0.2	84.3 ± 0.4	97.2	95.2	91	9,097,238	19.6	18.6
Ours	96.3 ± 0.2 *	85.1 ± 0.3 *	99.0	93.6	93	3,742,954	10.8	8.0
YOLOv13n	93.3 ± 0.3	80.7 ± 0.5	98.5	88.1	88	2,460,301	6.4	5.4

An asterisk (*) indicates that RCPNet is significantly better than the second-best model according to a paired *t*-test (*p* < 0.05).

**Table 10:** Comparison of deployment indicators between the baseline network and RCP-Net.

Module	GPU Memory (G)	Detection Times (ms)			Training Time (h)	FLOPs (G)	Size (MB)
		Pre-Processing	Inference	Post-Processing			
Baseline	2.96	0.1	0.4	1.1	0.369	6.0	5.4
RCPNet (ours)	2.98	0.1	0.4	1.3	0.383	10.8	8.0

## Data Availability

The original contributions presented in the study are included in the article. Further inquiries can be directed to the corresponding author.
